# Estimating the strength of soil stabilized with cement and lime at optimal compaction using ensemble-based multiple machine learning

**DOI:** 10.1038/s41598-024-66295-4

**Published:** 2024-07-03

**Authors:** Kennedy C. Onyelowe, Arif Ali Baig Moghal, Ahmed Ebid, Ateekh Ur Rehman, Shadi Hanandeh, Vishnu Priyan

**Affiliations:** 1 Department of Civil Engineering, Michael Okpara University of Agriculture, Umudike, Nigeria; 2 Department of Civil Engineering, University of the Peloponnese, 26334 Patras, Greece; 3 Department of Civil Engineering, Kampala International University, Kampala, Uganda; 4 Department of Civil Engineering, National Institute of Technology Warangal, Warangal, 506004 India; 5 Department of Civil Engineering, Faculty of Engineering, Future University in Egypt, New Cairo, Egypt; 6 Department of Industrial Engineering, College of Engineering, King Saud University, 11421 Riyadh, Saudi Arabia; 7 Department of Civil Engineering, Al-Balqa Applied University, As-Salt, Jordan; 8 Department of Civil Engineering, Louisiana Transportation Research Centre (LTRC), Louisiana State University, Baton Rouge, LA 70803 USA; 9 Department of Civil Engineering, SRM Institute of Science and Technology, Chennai, Tamil Nadu India

**Keywords:** Cohesive soil stabilization, Unconfined compressive strength, Cement, Lime, Optimal compaction, Machine learning classifier, Symbolic regression, Engineering, Materials science

## Abstract

It has been imperative to study and stabilize cohesive soils for use in the construction of pavement subgrade and compacted landfill liners considering their unconfined compressive strength (UCS). As long as natural cohesive soil falls below 200 kN/m^2^ in strength, there is a structural necessity to improve its mechanical property to be suitable for the intended structural purposes. Subgrades and landfills are important environmental geotechnics structures needing the attention of engineering services due to their role in protecting the environment from associated hazards. In this research project, a comparative study and suitability assessment of the best analysis has been conducted on the behavior of the unconfined compressive strength (UCS) of cohesive soil reconstituted with cement and lime and mechanically stabilized at optimal compaction using multiple ensemble-based machine learning classification and symbolic regression techniques. The ensemble-based ML classification techniques are the gradient boosting (GB), CN2, naïve bayes (NB), support vector machine (SVM), stochastic gradient descent (SGD), k-nearest neighbor (K-NN), decision tree (Tree) and random forest (RF) and the artificial neural network (ANN) and response surface methodology (RSM) to estimate the (UCS, MPa) of cohesive soil stabilized with cement and lime. The considered inputs were cement (C), lime (Li), liquid limit (LL), plasticity index (PI), optimum moisture content (OMC), and maximum dry density (MDD). A total of 190 mix entries were collected from experimental exercises and partitioned into 74–26% train-test dataset. At the end of the model exercises, it was found that both GB and K-NN models showed the same excellent accuracy of 95%, while CN2, SVM, and Tree models shared the same level of accuracy of about 90%. RF and SGD models showed fair accuracy level of about 65–80% and finally (NB) badly producing an unacceptable low accuracy of 13%. The ANN and the RSM also showed closely matched accuracy to the SVM and the Tree. Both of correlation matrix and sensitivity analysis indicated that UCS is greatly affected by MDD, then the consistency limits and cement content, and lime content comes in the third place while the impact of (OMC) is almost neglected. This outcome can be applied in the field to obtain optimal compacted for a lime reconstituted soil considering the almost negligible impact of compactive moisture.

## Introduction

Cohesive soils are a type of soil characterized by its cohesive properties, which are primarily due to the presence of clay minerals^1^. Unlike non-cohesive soils such as sand and gravel, which rely on particle friction for stability, cohesive soils possess cohesive forces that bind the particles together^2^. These cohesive forces result in cohesive strength, making cohesive soils generally more stable and less prone to erosion than non-cohesive soils^3^. Clay Content: Cohesive soils typically have a high clay content, consisting of fine particles with diameters less than 0.002 mm. Clay minerals such as kaolinite, illite, and montmorillonite are commonly found in cohesive soils^3^. Plasticity: Cohesive soils exhibit plastic behavior, meaning they can be molded or shaped when moist and retain their shape when dried^4^. This property is attributed to the presence of clay minerals, which can absorb water and undergo volume changes. Consolidation: Cohesive soils undergo consolidation when subjected to loads over time^5^. The water within the soil matrix is gradually expelled, leading to compression and settlement of the soil layers^6^. Shear Strength: Cohesive soils have both cohesion and frictional resistance, contributing to their shear strength^3^. The cohesive strength arises from the electrochemical bonds between clay particles, while the frictional resistance occurs between soil particles and is influenced by factors such as particle size, shape, and orientation^4^. Sensitivity to Water: Cohesive soils are highly sensitive to changes in moisture content^7^. When saturated with water, they can exhibit significant volume changes, leading to swelling and reduced strength^8^. Conversely, when dried, they can shrink and become susceptible to cracking and fissuring. Engineering Challenges: Cohesive soils pose unique engineering challenges due to their plasticity, sensitivity to water, and consolidation behavior. Proper site characterization, soil stabilization techniques, and engineering measures such as drainage and soil reinforcement are often required to mitigate the risks associated with cohesive soils in construction projects^9^. The common engineering applications of cohesive soils include: Foundations: Cohesive soils provide a stable base for building foundations, although proper foundation design and construction techniques are essential to account for their compressibility and swelling behavior^6^. Embankments and Slopes: Cohesive soils are often used in embankments and slopes for infrastructure projects such as highways, railways, and levees^2^. Slope stability analysis and erosion control measures are necessary to prevent slope failures and soil erosion. Excavation Support: Cohesive soils can be excavated for construction projects such as trenches, basements, and tunnels^5^. Temporary support systems such as shoring, sheet piles, and soil stabilization techniques are employed to prevent collapse during excavation. Landfills: Cohesive soils are used as liner materials and cover systems in landfill engineering to contain and isolate waste materials^10^. Geosynthetic liners and drainage systems are implemented to enhance the performance and environmental protection of landfill facilities^9^. Overall, cohesive soils play a critical role in civil engineering and construction projects, and understanding their properties and behavior is essential for successful project planning, design, and implementation^1^.

The Unconfined Compressive Strength (UCS) of cohesive soil stabilized with cement and lime at optimal compaction is an important parameter in geotechnical engineering^3^. It indicates the ability of the stabilized soil to withstand compressive loads without failure and is often used to assess the effectiveness of soil stabilization techniques^11^. The UCS of cohesive soil stabilized with cement and lime at optimal compaction can be determined through various methods as contained in the literature. Sample Preparation: Obtain undisturbed or disturbed soil samples representative of the site conditions^8^. Mix the cohesive soil with the appropriate proportions of cement and lime based on the desired stabilization ratio^7^. The stabilization ratio depends on factors such as soil type, moisture content, and engineering requirements. Thoroughly mix the soil, cement, and lime using appropriate equipment such as a mixer or rotary tiller to ensure uniform distribution of the stabilizers throughout the soil matrix^4^. Compaction: Compact the soil-stabilizer mixture using a standard compaction method such as Proctor compaction or modified Proctor compaction^9^. Optimize the compaction effort (e.g., compactive effort, moisture content) to achieve the maximum dry density and optimum moisture content for the stabilized soil mixture. This is typically determined through laboratory compaction tests^7^. Curing: After compaction, allow the stabilized soil specimens to cure under controlled conditions (e.g., room temperature, humidity) for a specified curing period^6^. The curing period depends on factors such as the type and dosage of stabilizers used and the desired strength development^12^. UCS Testing: After the curing period, conduct unconfined compression tests on the stabilized soil specimens to determine their UCS. Place the specimen in a uniaxial compression testing machine and apply axial load at a constant rate until failure occurs^12^. Measure the maximum compressive stress at failure, which represents the UCS of the stabilized soil. Analysis: Analyze the UCS results to assess the effectiveness of the cement and lime stabilization on the cohesive soil^5^. Compare the UCS values of stabilized soil specimens with un-stabilized soil specimens or with project specifications to evaluate the improvement in strength achieved through stabilization^11^. Quality Control and Assurance: Implement quality control measures throughout the stabilization process to ensure consistency and reliability of results^12^. Monitor key parameters such as soil moisture content, stabilizer dosage, compaction effort, and curing conditions to optimize the stabilization process and achieve desired strength characteristics^13^. By following these steps, engineers and geotechnical professionals can determine the UCS of cohesive soil stabilized with cement and lime at optimal compaction and assess its suitability for various construction applications, such as road subgrades, embankments, and foundation support.

Furthermore, cohesive soils, such as clay and silt, present unique challenges, practical importance, and applications in various fields. Cohesive soils often exhibit high plasticity, meaning they undergo significant deformation under load and are prone to swelling and shrinkage with changes in moisture content^6^. This can pose challenges for construction projects, as it may lead to settlement, heave, or instability of structures. Cohesive soils typically have low permeability, limiting their ability to drain water effectively. This can result in poor drainage, waterlogging, and increased risk of foundation instability or slope failure^13^. Changes in moisture content can significantly affect the engineering properties of cohesive soils. Wetting and drying cycles can lead to volume changes, loss of strength, and changes in soil structure, complicating construction and maintenance activities^1^. Excavating and handling cohesive soils can be challenging due to their cohesive nature and tendency to stick together. Specialized equipment and techniques may be required to mitigate issues such as soil sticking to machinery and trench collapse. Understanding the behavior of cohesive soils is crucial for designing safe and stable foundations for buildings, bridges, roads, and other infrastructure^5^. Proper site investigation, soil characterization, and geotechnical analysis are essential for assessing foundation bearing capacity, settlement, and slope stability. Cohesive soils play a significant role in geotechnical engineering projects, including earthworks, embankments, retaining walls, and landslide mitigation. Proper soil stabilization techniques and drainage solutions are often required to ensure the stability and durability of these structures^12^. Cohesive soils can act as barriers to groundwater flow and play a role in controlling contaminants and pollutants in the subsurface. Understanding the permeability and hydraulic conductivity of cohesive soils is essential for groundwater remediation, landfill design, and environmental protection^3^. Cohesive soils are also used as construction materials in certain applications, such as in the manufacture of clay bricks, ceramics, and earthen construction techniques like rammed earth and adobe. Cohesive soils are commonly encountered in road construction projects, where they are used as subgrade materials or as part of engineered fills^4^. Proper compaction and stabilization techniques are essential to ensure the durability and performance of roadways. Cohesive soils are often used in land reclamation projects to reclaim and stabilize land for development purposes, such as building new residential or industrial areas on reclaimed land^7^. Understanding the properties of cohesive soils is critical for agricultural practices, as they influence soil fertility, drainage, and crop productivity. Proper soil management techniques, such as tillage, drainage, and irrigation, are essential for maximizing agricultural yields in cohesive soil areas^9^. Cohesive soils are used in the construction of engineered landfills and containment facilities for storing hazardous and non-hazardous waste^14^. The low permeability of cohesive soils helps prevent the migration of contaminants into the surrounding environment. Overall, cohesive soils present challenges in construction, engineering, and environmental applications, but they also offer opportunities for innovative solutions and sustainable development especially as a rconstituted construction material as applied in this case where cement and lime have been utilized in the stabilization of the soil for a more sustaining construction of subgrade and landfill liners. Understanding the behavior and properties of cohesive soils is essential for addressing these challenges and leveraging their practical importance in various fields.

## Literature reviews

Ghanizadeh et al.^13^ used the evolutionary polynomial regression (EPR) technique to forecast the compressive strength and Young's modulus of lime/cement stabilized clayey subgrade soil. Specimens with varying cement and lime concentrations were subjected to different curing durations. The R^2^ values of the model for predicting UCS were 0.96 and 0.95 for cement-stabilized clay soil, 0.91 and 0.87 for lime-stabilized soil, and 0.88 and 0.94 for lime-stabilized soil. The study found that both Portland cement and moisture content were crucial factors affecting the Unconfined Compressive Strength (UCS) and Young's modulus. Onyelowe et al.^15^ used Gene expression programming (GEP) and multi-expression programming (MEP) for the prediction of the unconfined compressive strength of soil under unsaturated situations. These models are essential for designing the subgrade of flexible pavements because laboratory estimates are complex and time-consuming. The soil underwent treatment using hybrid cement, which is a composite binder, together with nanostructured quarry fines. A baseline regression model was performed to assess the extent of concordance between the target variable and its various predictors. The findings demonstrated that GEP surpassed MEP and MLR in terms of performance. GEP achieved a coefficient of determination (R^2^) of 0.99 and 0.988, a relative root-mean-square error (RRMSE) of 0.0728 and 0.0741, and proximal performance indicators of 0.036 and 0.037 for training and validation, respectively. Mamat and Ramli^16^ investigated the impact of different lime percentages on the strength of peat. The findings indicate that the Evolutionary Polynomial Regression (EPR) model, using a hyperbolic tangent function, is the most accurate in predicting the unconfined compressive strength values. This model can be utilized to ascertain the most favorable quantity of stabilizers for projects aimed at enhancing soft ground conditions. Consequently, it enhances the accuracy of estimating the required amount of lime for improving peat ground. Jurong^17^ examined the impact of different mix proportions and material types on the rate at which cement stabilizes inorganic clayey soil and increases its strength. The soil was categorized into two groups: fine-grained clay (with granular content of 10% or less) and clay with sand impurities (with granular content above 10%). The ratio of water to cement (w/c) significantly affects the development of strength, with the ratio of water to solids (w/s) having a bigger influence than the ratio of solids to cement (s/c) in most situations. The impact of fine-grained inorganic clay type is directly related to the liquid limit and activity of the clay, which enhances the pozzolanic reaction between clay particles and calcium hydroxide, leading to an increase in strength development. A unique strength prediction model was suggested, which utilizes the Cumulative Distribution Function (CDF) of a log-normal distribution. This model accurately matches the early-stage strength development with the theoretical ultimate strength. An investigation was conducted to examine the impact of temperature on the strength development of cement-stabilized clay including sand impurities. The results revealed that higher temperatures have a positive effect on the overall strength enhancement of clay with up to 60% sand impurities. However, a phenomenon known as 'cross-over' behavior was seen in clay samples with 80% sand impurities and 100% sand. Utilizing data-driven techniques, such as gradient enhanced trees, demonstrated encouraging efficacy in forecasting and elucidating the progress of strength growth in cement stabilized soils. Ghanizadeh, Safi Jahanshahi, and Naseralavi^18^ assessed machine learning algorithms to forecast the unconfined compressive strength of cement-stabilized iron ore tailings (IOT), with the goal of optimizing the procedure and minimizing expenses related to quality control. The artificial neural network method demonstrated exceptional accuracy in both training and testing data. The cement % was shown to have the most substantial influence on the unconfined compressive strength. A comprehensive analysis was conducted to assess the impact of several variables on the unconfined compressive strength. Shariatmadari et al.^14^ investigated the utilization of volcanic ash and powdered granulated blast furnace slag as substitute construction materials for the purpose of stabilizing sandy soils. Alkali activator solutions were employed, and samples underwent curing at different temperatures and durations. The unconfined compressive strength was assessed through the utilization of artificial neural network (ANN) modelling and evolutionary polynomial regression technique. A neural network model with an architecture of 8–5–10–1 was proposed to enhance the accuracy of predicting the unconfined compressive strength. The Si/Al ratio and VA content were identified as the most influential parameters based on the parametric research. Jalal et al.^19^ introduced novel empirical prediction models for assessing the swell pressure and unconfined compression strength of expansive soils through the utilization of artificial neural networks, adaptive neuro fuzzy inference system (ANFIS), and gene expression programming. A comprehensive database consisting of 168 Ps and 145 UCS records was created, with nine geotechnical factors included as predictor variables. The models underwent training and testing, and their performance was compared to the observed findings. The results demonstrated that both GEP and ANN are efficacious and dependable methods for estimating UCS. The GEP model exhibited superior performance compared to the other two models in terms of data proximity and optimal fit. These findings can assist researchers, designers, and practitioners in assessing the swell-strength properties of expansive soils, resulting in expedited, safer, and environmentally-friendly construction practices. Mamat and Ramli^16^ developed a predictive model for the unconfined compressive strength (UCS) of clay stabilization using the gene-expression programming technique. A total of eleven criteria were taken into account, encompassing factors such as soil conditions, types of binder, binder quantities, mixing method, and duration of curing. The GEP-based model achieved excellent performance, exhibiting a satisfactory correlation coefficient and few errors. The results of the parametric studies indicate that the plastic index, clay percentage, and water content have a detrimental impact on the unconfined compressive strength. Conversely, the silt and sand percentage, binder types, and curing duration have a beneficial influence on the strength. Onyelowe et al.^15^ utilized Multi-Gene Genetic Programming (MGGP) and Artificial Neural Network (ANN) methodologies to forecast the unconfined compression strength (UCS) of Queensland soil stabilized with cement/lime. The models assess the influence of the kind, quantity, and duration of binder on the unconfined compressive strength (UCS). The results demonstrate strong correlation coefficients and minimal errors, with curing time exerting the most prominent influence. The utilization of predictive equations can assist engineers and consultants in making informed decisions regarding the suitable binder and its optimal quantity during the pre-planning and design stages. Saadat and Bayat^20^ investigated the influence of the amount of stabilizer, duration of curing, and level of moisture on the Understanding the UCS of 150 soil samples that have been stabilized. The study determines that the lime or cement content is directly proportional to the maximum unconfined compressive strength, resulting in greater UCS values as the stabilizer content increases. This study indicated that the utilization of fuzzy logic and NLR can enhance the accuracy of UCS prediction. ANFIS, in particular, demonstrated a more favorable prediction performance with correlation values of 94% and 84%. Many other works have studied the machine learning application and sensitivity analysis of independent variables towards the optimal performance of engineering systems^21–35^ Also, Raja et al.^36^ explored the physics-based ML techniques to study the stochastic reliability modeling for reinforced soil foundation making use of multiple data cases generated using the constitutive relations of the foundation parameters. This gave further insight on the application of ML in solving soil stabilization problems by optimizing the failure case with the novel gene expression programming (GEP). From the extensive reviews, it can be found that different machine learning techniques have been deployed to predict the strength of clayey soils reconstituted with lime, cement and a combination of lime and cement. The results achieved from the reviews in this report can be compared to the present work, which has applied multiple machine learning techniques (nine of them) to study the behavior of the lime and cement reconstituted cohesive soil considering the optimal compaction state conditions. The robust application of the multiple machine learning allows for more open-ended consideration of more efficient techniques from more decisive models, which are to be deployed in designs and field applications. While the highest number of entries applied in the previous research projects is 150, the present research paper reports the application of 190 entries, which eventually produced more reliable models. It is important to also note that the machine learning techniques applied in the present work are symbolic techniques, which apart from the contribution of an increased number of data entries to their performance got improvement from the open-ended algorithmic interface that allowed their robust running and iterations.

## Methodology of research

### Preliminary statistics of the collected database

**Figure 1:**
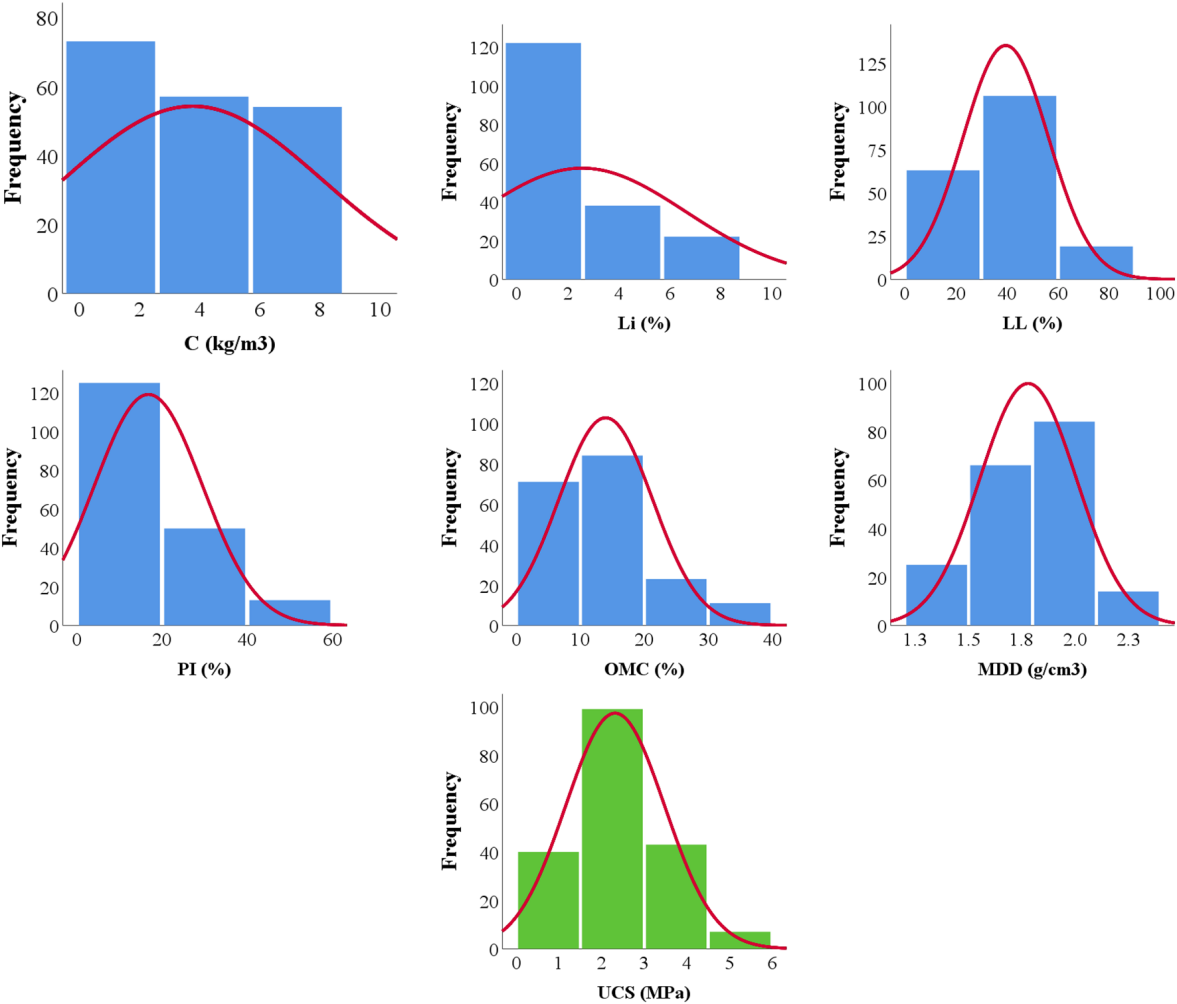
Distribution histograms for inputs (in blue) and outputs (in green).

**Figure 2:**
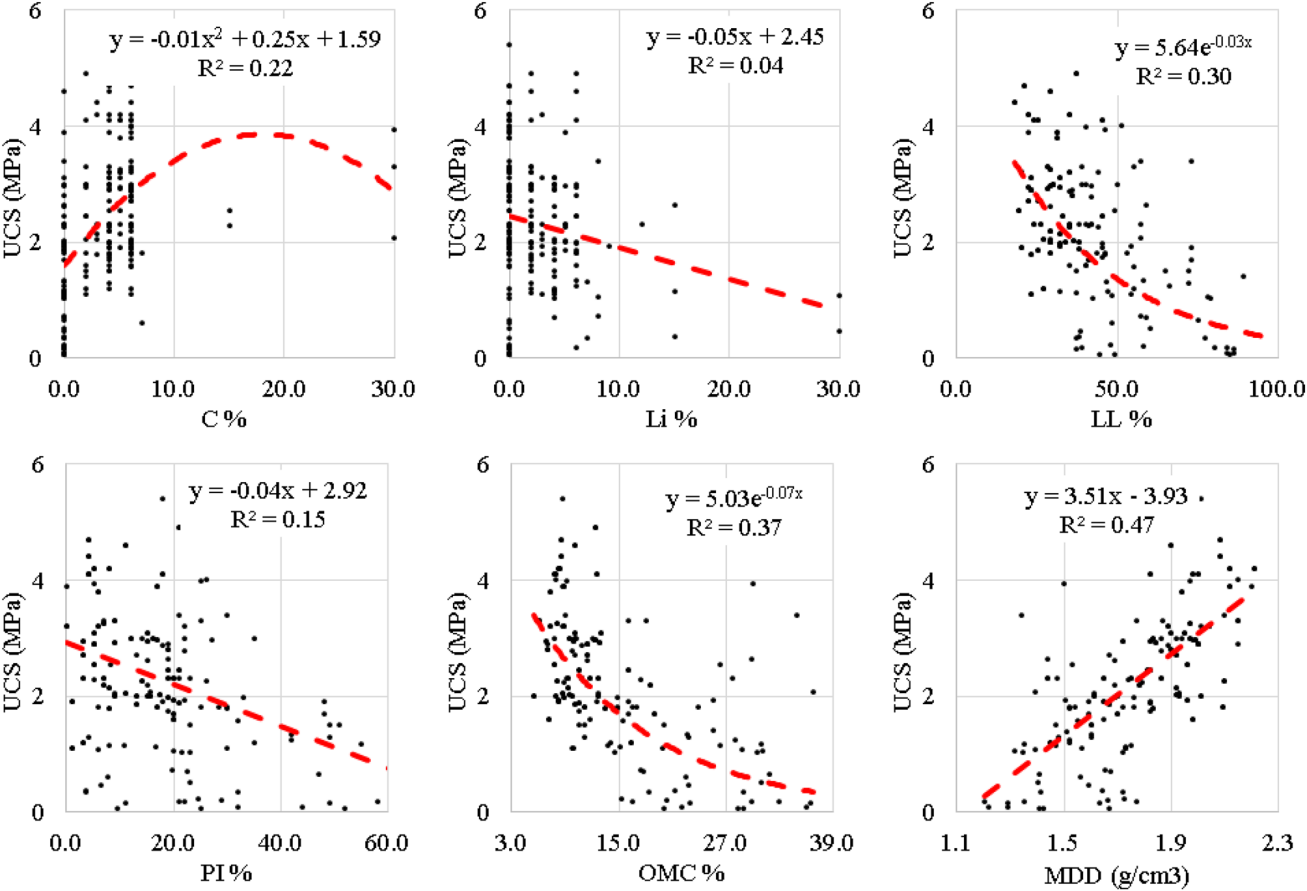
Relations between inputs and output.

A total of 190 records were collected from literature for UCS test results of cohesive soil stabilized with cement and lime. Each record contains the following data^1^: Cement weight ratio (C), %, Lime weight ratio (Li), %, Liquid limit (LL), %, Plasticity index (PI), %, Optimum moisture content (OMC), %, Maximum dry density (MDD), g/cm^3^, and Unconfined compressive strength (UCS), MPa. Data-preprocessing through data cleaning and dimensionality reduction methods was conducted to reduce the complexities in the models run. The collected records were divided into training set (140 records) and validation set (50 records). Tables 1 and 2 summarize their statistical characteristics and the Pearson correlation matrix. Finally, Fig. 1 shows the histograms for both inputs and outputs and Fig. 2 shows the relations between the inputs and the outputs.

**Table 1:** Statistical analysis of collected database.

	C	Li	LL	PI	OMC	MDD	UCS
	(%)	(%)	(%)	(%)	(%)	(g/cm^3^)	(MPa)
Training dataset							
Max	30.0	30.0	102.0	70.0	36.8	2.2	5.4
Min	0.0	0.0	18.0	0.0	5.4	1.2	0.1
Avg	4.0	2.5	39.9	17.5	14.2	1.8	2.4
SD	4.8	3.8	17.1	13.3	7.4	0.2	1.2
Var	1.2	1.5	0.4	0.8	0.5	0.1	0.5
Validation dataset							
Max	30.0	30.0	80.0	49.0	33.0	2.2	4.4
Min	0.0	0.0	18.0	1.0	6.9	1.2	0.1
Avg	3.3	2.6	38.4	14.7	13.1	1.8	2.3
SD	2.5	4.7	15.4	10.4	6.9	0.2	1.1
Var	0.8	1.8	0.4	0.7	0.5	0.1	0.5

**Table 2:**
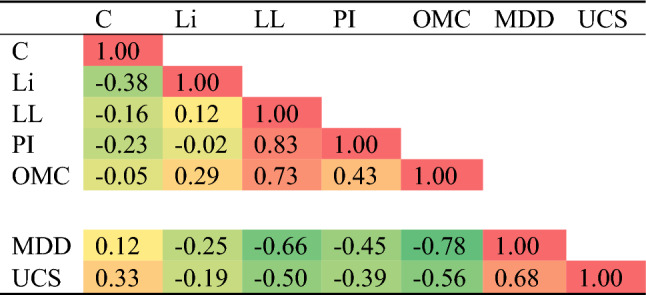
Pearson correlation matrix.

Table 1 presents the statistical characteristics for both training and validation sets in terms of mean, range, standard deviation and variance of each parameter. The main goal of this table is to assure that both sets have the same statistical characteristics and hence, validation set could be used to monitor the learning process and stop the training before over-fitting. While Table 2 presents the correlation between each parameter, there are two aims for this table, the first is to insure that all considered parameters are independent (correlation factor < 0.9), the second is to rank the inputs according to their correlation (importance) to the output.

Figure 1, shows the histogram of each parameter, it used to insure that there is no “gaps” in the distribution of any parameters because predictive model should be trained using the considered full range of each parameter. Gaps in the distribution may lead to inaccurate predictions in these zones. The histograms in Fig. 1 showed that there is no bias in (C, LL, OMC & MDD) and although there is lift shifting in (PI & Li) but the output (UCS) still unbiased.

Finally, Fig. 2 presents the relations between the output and each input, it is another graphical alternative to the correlation matrix presented in Table 2. The fitting line in red gives a general idea about the relation (forward or revers) while the (R^2^) evaluates the strength of this relation.

### Research program

**Figure 3:**
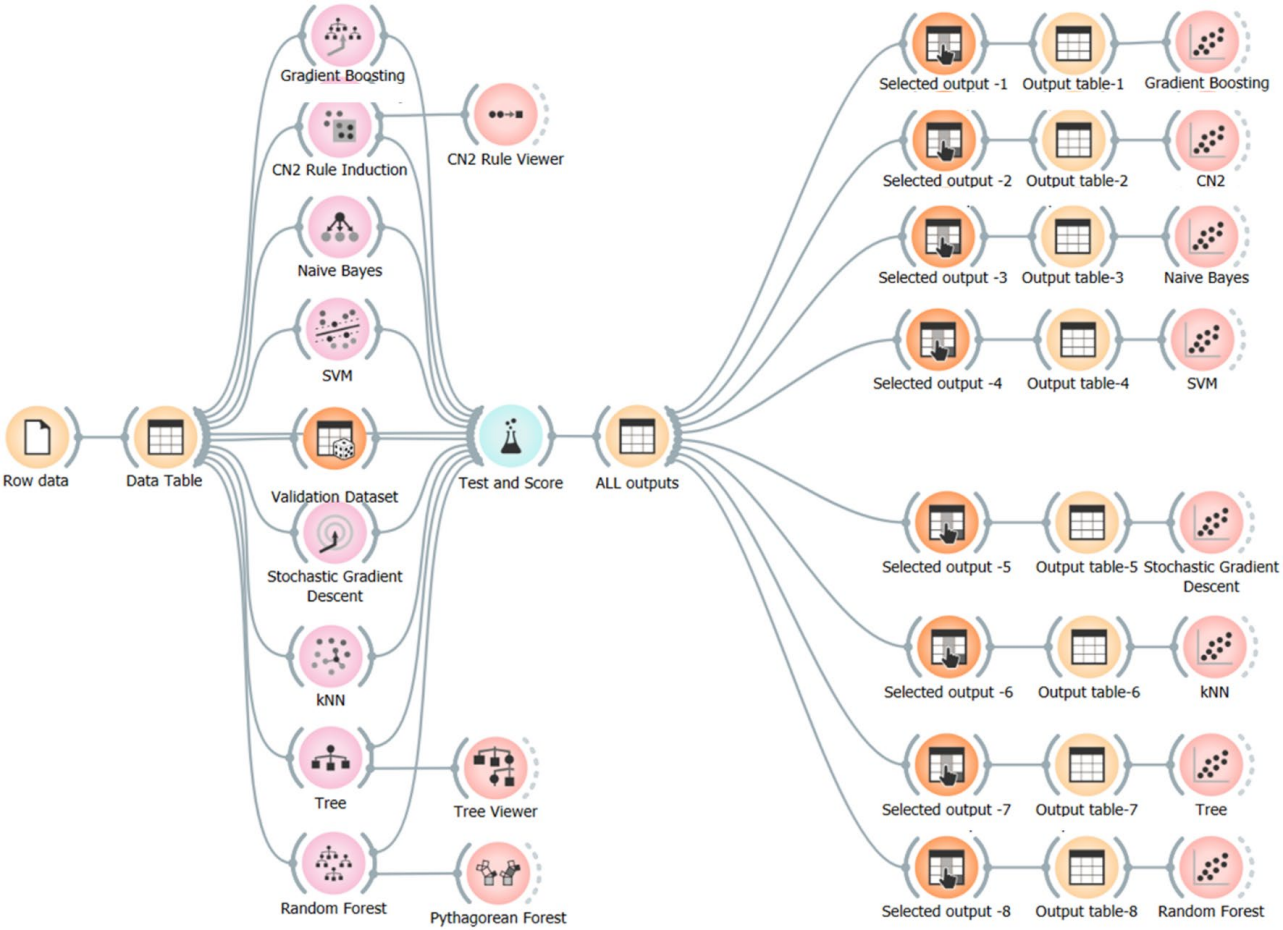
The considered data flow in Orange software.

Eight different ML classification techniques were used to predict the (UCS) of stabilized soil using the collected database. These techniques are “Gradient Boosting (GB)”, “CN2 Rule Induction (CN2)”, “Naive Bayes (NB)”, “Support vector machine (SVM), “Stochastic Gradient Descent (SGD)”, “K-Nearest Neighbors (KNN)”, “Tree Decision (Tree)” and “Random Forest (RF)”. All the eight developed models were created using used to predict (UCS) using (C), (Li), (LL), (PI), (OMC) and (MDD). All the developed models were created using “Orange Data Mining” software version 3.36. The considered data flow diagram is shown in Fig. 3. The flow began with reading the database and store it in a table, then the validation set is splatted in a separate table. Copies from the training table is introduced to the training modules of the considered techniques, graphical presentations are generated for the developed models that have ones such as CN2, Tree & RF.

Both training and validation sets are evaluated for all techniques in the node called “Test and score” the predicted values for both training and validation sets for all techniques are stored in table called “All outputs” which feeds the presentation generators. For each technique, the related outputs are selected from the “All outputs” table and stored in a separate table and the relation between predicted and experimental values is plotted.

### Decision-based classifier and ensemble machine learning techniques

#### Gradient boosting (GB)

**Figure 4:**
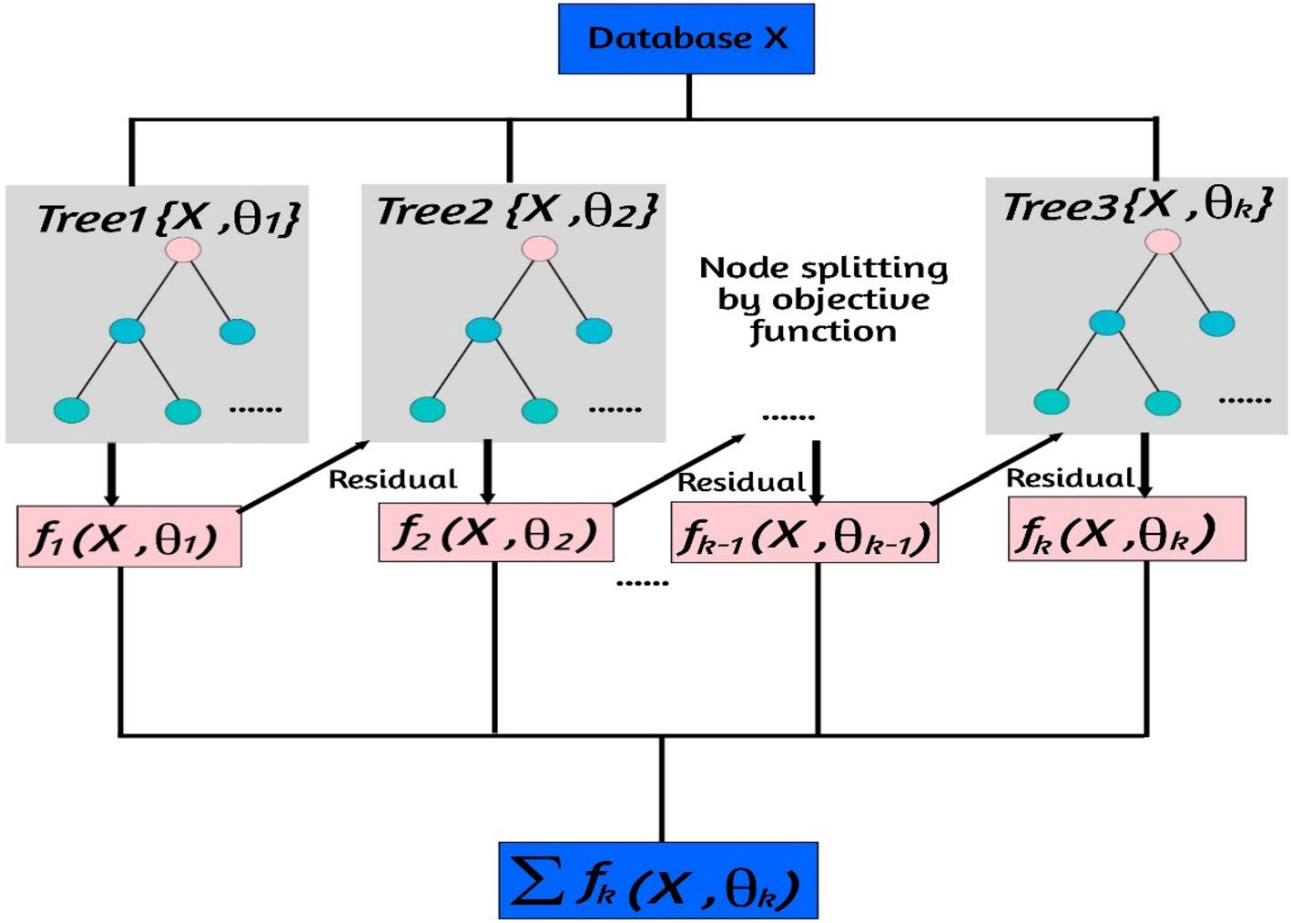
GB framework.

Gradient boosting is a popular machine learning technique used for both regression and classification tasks^15^. The GB framework is presented in Fig. 4. It is an ensemble method that combines multiple weak predictive models, typically decision trees, to create a strong predictive model. The basic idea behind gradient boosting is to iteratively add weak models to the ensemble, with each subsequent model aiming to correct the mistakes made by the previous models^6^. This is done by minimizing a loss function, which measures the difference between the predicted and actual values^15^. Here's a step-by-step overview of how gradient boosting works: Initialize the ensemble model by creating the first weak model, often a shallow decision tree. Fit the weak model to the training data and calculate the residuals, which are the differences between the predicted and actual values. Train a new weak model on the residuals. This model is designed to predict the residuals based on the input features^3^. Add the new model to the ensemble by combining it with the previous models, weighted by a learning rate. The learning rate determines the contribution of each model to the final prediction. Repeat steps 2 to 4 for a specified number of iterations or until a stopping criterion is met. In each iteration, the new weak model is trained to predict the negative gradient of the loss function with respect to the ensemble's predictions^2^. Finally, the ensemble model is formed by combining the predictions of all the weak models, weighted by their respective learning rates. Gradient boosting has several advantages: It can handle different types of data, including categorical and numerical features. It can capture complex interactions between variables and automatically handle feature interactions. It typically produces highly accurate predictions, as the ensemble model improves with each iteration. It is less prone to overfitting compared to other algorithms like decision trees. However, gradient boosting also has some considerations: It can be computationally expensive, especially when dealing with large datasets or complex models^15^. It requires careful tuning of hyperparameters such as the number of iterations, learning rate, and tree depth. It may be sensitive to noisy or irrelevant features, which can lead to overfitting. Overall, gradient boosting is a powerful technique that has been successfully applied in various domains, including finance, healthcare, and natural language processing. It is implemented in popular machine learning libraries such as XGBoost, LightGBM, and CatBoost.

#### CN2 rule induction (CN2)

**Figure 5:**
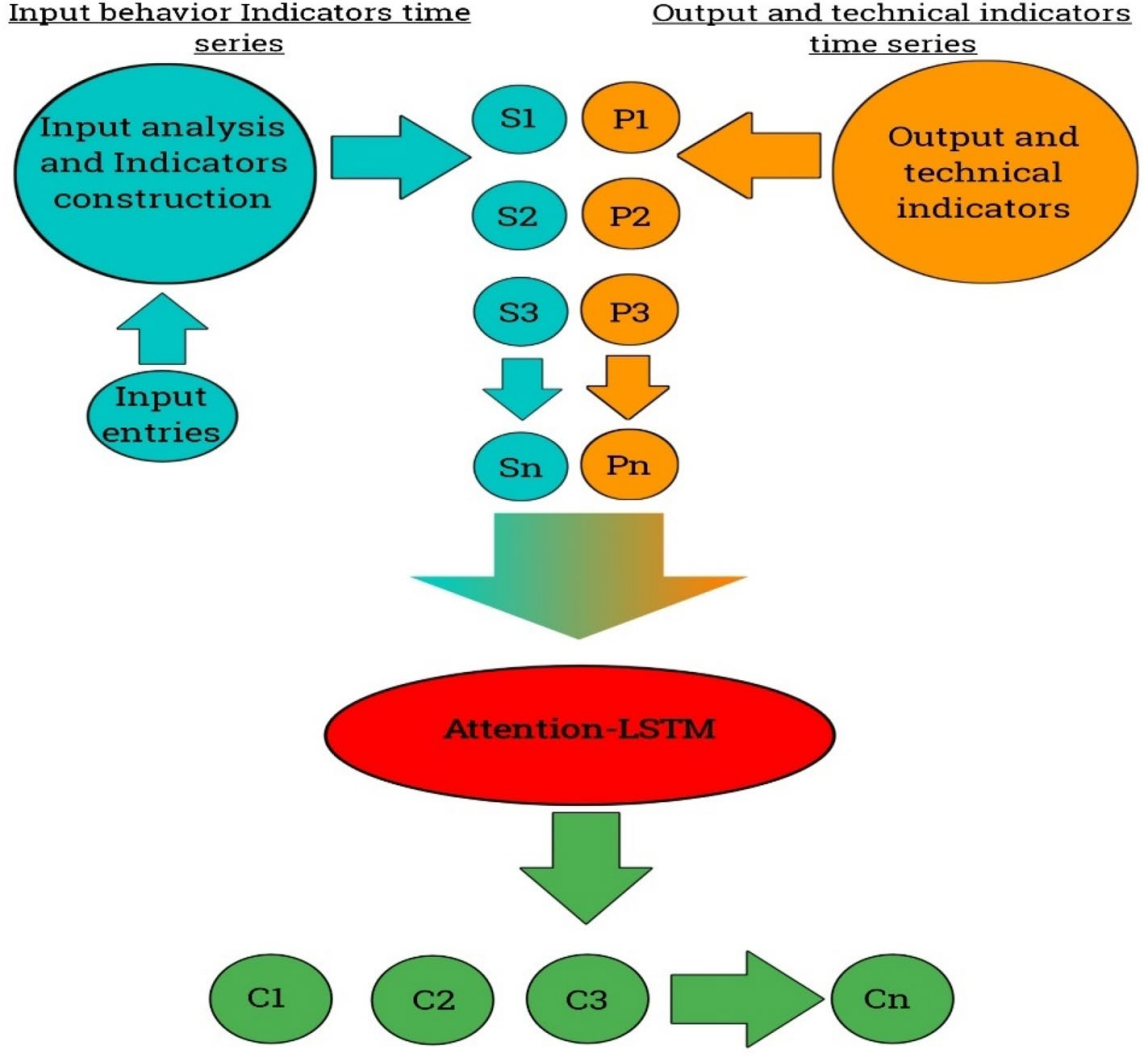
CN2 framework.

CN2 (Conceptual Clustering on Numerical Data) is a rule induction algorithm used for classification tasks^15^. The CN2 framework is presented in Fig. 5. It is designed to discover compact and accurate sets of classification rules from numerical data. The CN2 algorithm follows a top-down, greedy search approach to build a set of rules that collectively describe the data. Here's a step-by-step overview of how CN2 works: Initialize an empty set of rules. Select a target class and find the best rule for that class^2^. The best rule is the one that maximizes a quality measure, such as information gain or accuracy, when applied to the current dataset. Prune the instances covered by the best rule from the dataset. Repeat steps 2 and 3 until a stopping criterion is met. This could be a maximum rule length, a minimum number of instances covered by each rule, or a predefined number of rules. Optionally, post-process the generated rules to remove redundant or conflicting rules. The resulting set of rules can be used to classify new instances by sequentially applying each rule and assigning the instance to the target class of the first matching rule. CN2 has some key characteristics and advantages: It is well-suited for datasets with numerical attributes, as it handles them naturally. It can discover interpretable rules that describe the underlying patterns in the data. CN2 is a deterministic algorithm that produces consistent results^21^. It is relatively efficient compared to other rule induction algorithms. However, there are also some considerations with CN2: CN2 is a top-down algorithm, which means it may miss some rules that could be discovered by a bottom-up approach. It assumes that each instance can be classified by a single rule, which may not always be the case in complex datasets^6^. The performance of CN2 heavily depends on the quality of the quality measure used to select the best rule. Choosing an appropriate quality measure is crucial. The CN2 algorithm has been used successfully in various domains, including medicine, finance, and customer relationship management. It provides a transparent and interpretable way to extract knowledge from numerical data.

#### Naive Bayes (NB)

**Figure 6:**
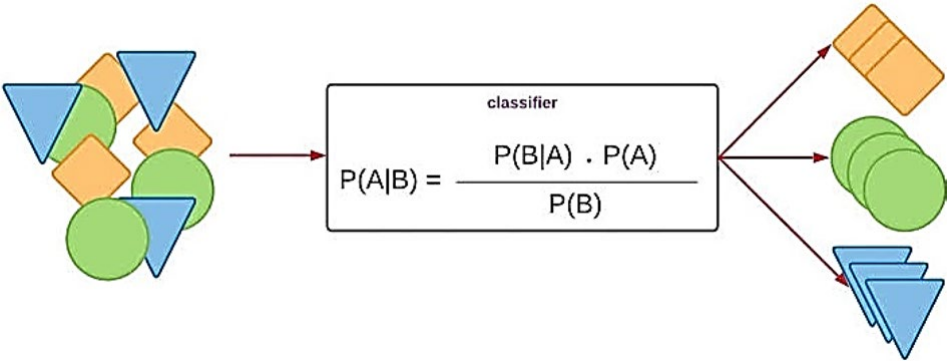
NB framework.

Naive Bayes is a popular machine learning algorithm for classification tasks. The NB framework is presented in Fig. 6. It is based on Bayes' theorem and makes the assumption of attribute independence, which is why it is called "naive". Despite this simplifying assumption, Naive Bayes has been proven to be effective in many real-world applications and is known for its simplicity, speed, and ability to handle large feature spaces^15^. Here's an overview of how the Naive Bayes algorithm works: Data Preparation: Prepare a labeled dataset where each instance consists of a set of features (attributes) and a corresponding class label. The features can be categorical or numerical. Probability Estimation: Calculate the prior probabilities of each class, which represent the probability of encountering each class in the dataset. This is done by counting the occurrences of each class in the training set. Feature Probability Estimation: For each feature, estimate the conditional probability of that feature given each class. This is typically done differently depending on whether the feature is categorical or numerical^6^. Categorical Features: Calculate the probability of each category occurring within each class by counting the occurrences of each category in the training set. Numerical Features: For each class, estimate the mean and standard deviation of the feature values. Assuming a Gaussian distribution, use these statistics to calculate the probability density function of the feature value given the class. Classification: Given a new instance with unknown class, calculate the posterior probability of each class given the instance's features using Bayes' theorem. The class with the highest posterior probability is assigned as the predicted class for the instance. Naive Bayes has several advantages: It is computationally efficient and can handle large datasets and high-dimensional feature spaces. The assumption of attribute independence makes it robust and requires fewer training instances compared to other algorithms. It can handle both categorical and numerical features^15^. Naive Bayes is relatively less prone to overfitting, as long as the independence assumption holds reasonably well. However, there are some considerations with Naive Bayes: The assumption of attribute independence may not hold in some cases, leading to suboptimal results. It can be sensitive to the presence of irrelevant or redundant features. Naive Bayes tends to underestimate the probability of rare events due to the assumption of attribute independence. Despite these limitations, Naive Bayes is widely used in various domains, including text classification, spam filtering, sentiment analysis, and recommendation systems. It serves as a simple and effective baseline algorithm for many classification tasks.

#### Support vector machine (SVM)

**Figure 7:**
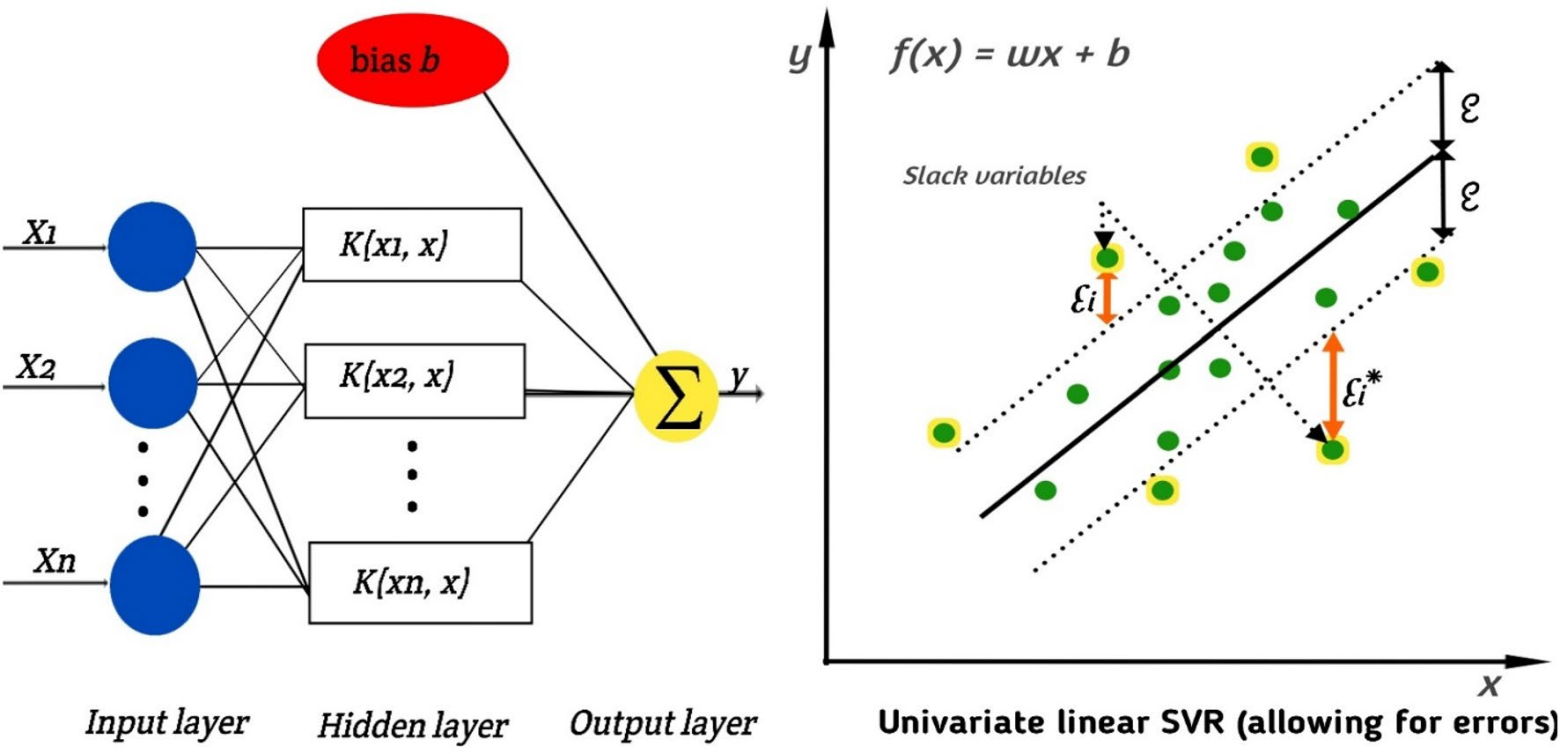
SVM framework.

Support Vector Machines (SVMs) are a powerful and versatile supervised learning algorithm used for classification and regression tasks^21^. The SVM framework is presented in Fig. 7. They are particularly effective in handling complex, high-dimensional datasets. The main idea behind SVMs is to find an optimal hyperplane that separates different classes in the feature space. The hyperplane is selected in such a way that it maximizes the margin, which is the distance between the hyperplane and the nearest data points of each class. The data points closest to the hyperplane are called support vectors ^3^. Here's an overview of how the Support Vector Machine algorithm works for binary classification: Data Preparation: Prepare a labeled dataset where each instance consists of a set of features (attributes) and a corresponding class label. Feature Mapping: Transform the input features into a higher-dimensional space using a kernel function. This allows SVMs to find nonlinear decision boundaries in the original feature space. Hyperplane Selection: Find the optimal hyperplane that maximizes the margin between the classes. This can be done by solving an optimization problem that involves minimizing the classification error and maximizing the margin. Soft Margin Classification: In cases where the data is not perfectly separable, SVMs can allow some misclassifications by introducing a slack variable^21^. This leads to a soft margin that allows for a trade-off between maximizing the margin and minimizing the misclassification errors. Kernel Trick: SVMs leverage the kernel trick, which avoids the explicit computation of the high-dimensional feature space. Instead, it uses a kernel function to calculate the inner products between the transformed feature vectors, making the computations efficient. Classification: To classify new instances, the algorithm computes the distances between the new instances and the hyperplane. The predicted class is determined by the side of the hyperplane on which the new instance falls. SVMs have several advantages: They can handle high-dimensional feature spaces effectively. SVMs are robust against overfitting, especially when using a proper regularization parameter. The kernel trick allows SVMs to capture complex nonlinear relationships in the data. SVMs have a solid theoretical foundation and are well-studied^3^. However, there are some considerations with SVMs: SVMs can be computationally expensive, especially for large datasets. SVMs require proper tuning of hyperparameters, such as the choice of kernel function and regularization parameter. SVMs can be sensitive to outliers in the data. SVMs have been successfully applied in various domains, including text classification, image recognition, and bioinformatics.

#### Stochastic gradient descent (SGD)

**Figure 8:**
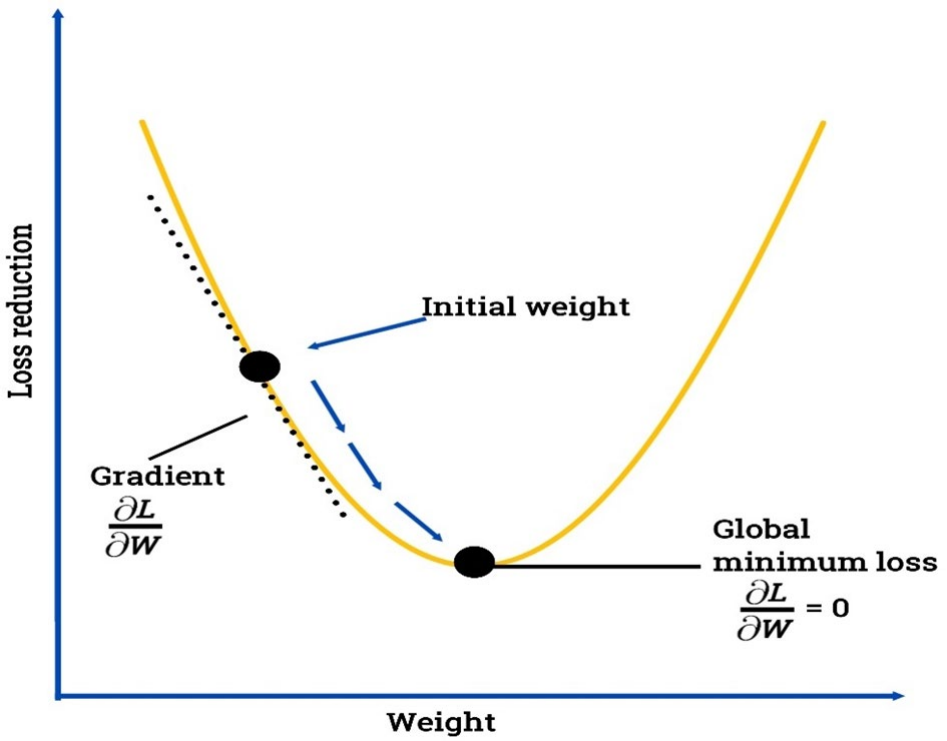
SGD framework.

Stochastic Gradient Descent (SGD) is an optimization algorithm commonly used for training machine learning models, particularly in large-scale settings^6^. The SGD framework is presented in Fig. 8. It is an iterative algorithm that updates the model parameters based on the gradients of the loss function estimated from a randomly sampled subset of the training data. Here's an overview of how Stochastic Gradient Descent works: Data Preparation: Prepare a labeled dataset with the input features and corresponding target labels^6^. Model Initialization: Initialize the model's parameters, such as the weights and biases, with random values or predefined values. Random Shuffling: Shuffle the training data randomly. This step is typically performed in each epoch to ensure a different ordering of the data in each iteration. Iterative Update: Iterate through the training data in small batches, known as mini-batches, rather than using the entire dataset at once. This is the "stochastic" part of SGD. Gradient Calculation: For each mini-batch, calculate the gradients of the loss function with respect to the model parameters. These gradients indicate the direction and magnitude of the parameter updates. Parameter Update: Update the model parameters using the calculated gradients^15^. The update is performed in the opposite direction of the gradients, scaled by a learning rate. The learning rate determines the step size taken in the parameter space. Repeat Steps 4 to 6: Iterate through the mini-batches for a specified number of epochs or until a convergence criterion is met. Each iteration updates the model parameters based on a different subset of the training data. SGD has some advantages: It is computationally efficient and memory-friendly, as it processes the data in mini-batches rather than the entire dataset. SGD can handle large-scale datasets, making it suitable for scenarios with a massive amount of training data. It can continually update the model parameters as new data becomes available, making it suitable for online learning scenarios^21^. However, there are also considerations with SGD: SGD is sensitive to the learning rate. Choosing an appropriate learning rate can be crucial for convergence and model performance. The algorithm may get stuck in suboptimal solutions or saddle points, particularly for non-convex loss functions. The random sampling of mini-batches can introduce noise, which can affect the convergence and stability of the optimization process. Variations of SGD, such as mini-batch SGD and momentum-based SGD, have been proposed to address some of the limitations and improve convergence speed. SGD is widely used as the optimization algorithm for training various machine learning models.

#### K-nearest neighbors (K-NN)

**Figure 9:**
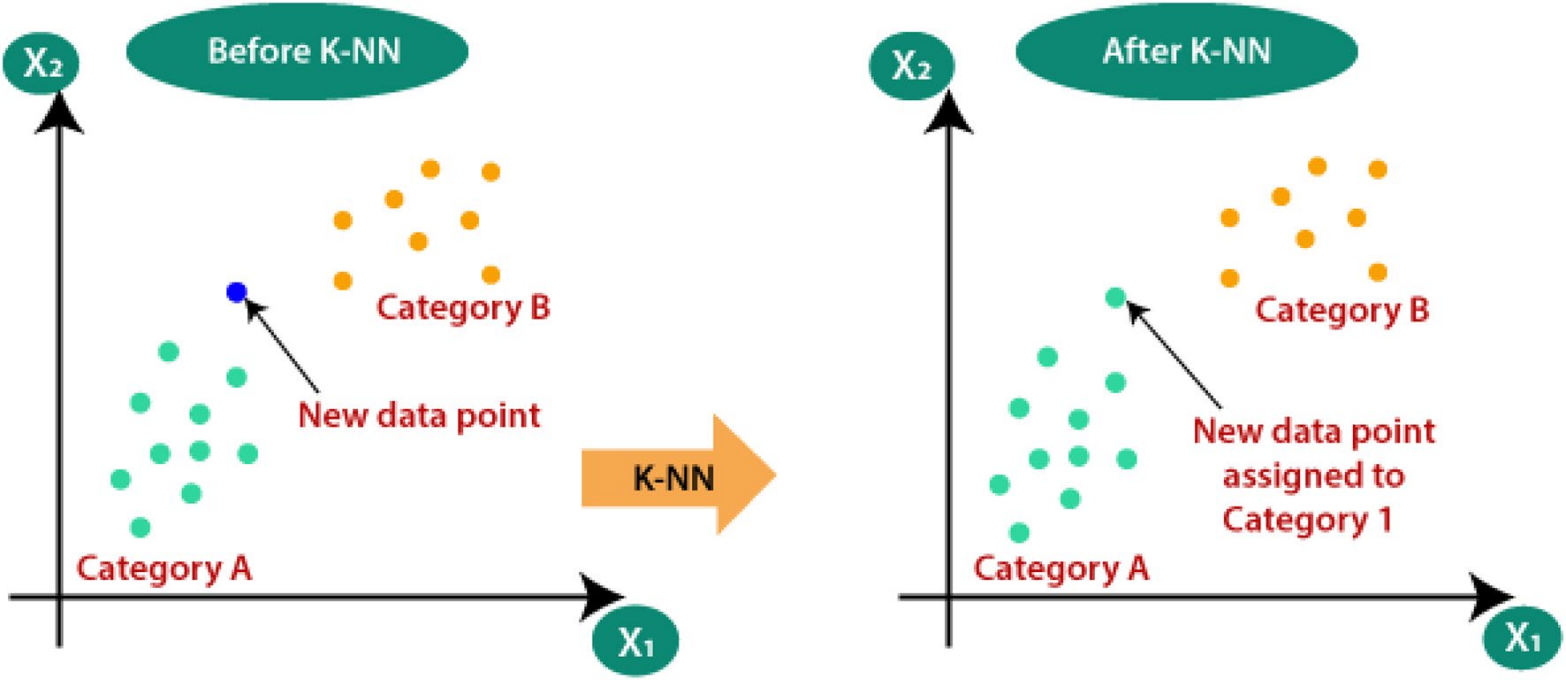
K-NN framework.

K-nearest neighbors (K-NN) is a popular supervised learning algorithm used for both classification and regression tasks^15^. The K-NN framework is presented in Fig. 9. It is a non-parametric algorithm that makes predictions based on the similarity of the training instances to the new, unseen instances. Here's an overview of how the K-nearest neighbors algorithm works for classification: Data Preparation: Prepare a labeled dataset where each instance consists of a set of features (attributes) and a corresponding class label^3^. Select the Value of K: Choose a value for K, which represents the number of nearest neighbors to consider when making predictions. The value of K is typically determined through experimentation or cross-validation. Distance Calculation: Calculate the distance between the new instance and each instance in the training set. The most commonly used distance metric is Euclidean distance, but other metrics like Manhattan distance or cosine similarity can also be used. Nearest Neighbor Selection: Select the K instances with the smallest distances to the new instance. These instances are called the K nearest neighbors. Majority Voting: For classification, determine the class label of the new instance based on the class labels of its K nearest neighbors. The class label with the highest frequency among the neighbors is assigned as the predicted class label. K-NN has several characteristics: K-NN is a lazy learner, meaning it does not build an explicit model during training. Instead, it memorizes the training instances and performs calculations at prediction time. It can handle both categorical and numerical features. K-NN can capture complex decision boundaries and is capable of nonlinear classification. The choice of K affects the algorithm's performance. Smaller values of K can lead to more flexible decision boundaries, but they are more sensitive to noise and outliers. Larger values of K provide smoother decision boundaries but may lead to over-smoothing. K-NN does not make any assumptions about the underlying data distribution, making it versatile and applicable to various domains^6^. However, there are considerations with K-NN: K-NN can be computationally expensive, especially when dealing with large datasets or high-dimensional feature spaces. Efficient data structures, such as KD-trees or ball trees, are often used to speed up the search for nearest neighbors. The algorithm is sensitive to the choice of distance metric. Different metrics may perform better or worse depending on the data characteristics. K-NN may struggle with imbalanced datasets, as the majority class can dominate the prediction for nearby instances. K-NN is widely used in various domains, such as recommendation systems, image recognition, and anomaly detection.

#### Tree decision (tree)

**Figure 10:**
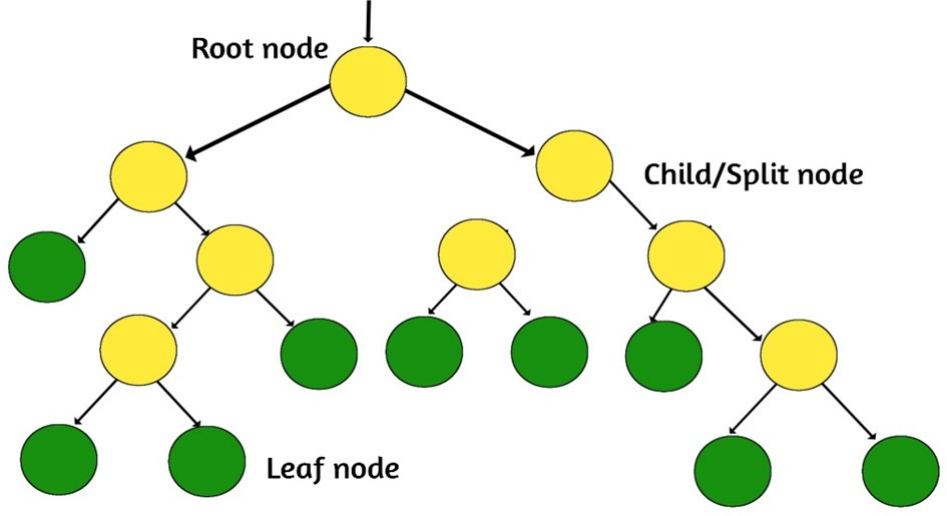
Tree framework.

Decision trees are a popular machine learning algorithm used for both classification and regression tasks^2^. The Tree framework is presented in Fig. 10. They are simple yet powerful models that make predictions by recursively partitioning the feature space based on the values of input features. Here's an overview of how decision trees work: Data Preparation: Prepare a labeled dataset where each instance consists of a set of features (attributes) and a corresponding class label or target value. Tree Construction: The decision tree algorithm starts by selecting the best feature to split the dataset. This is typically done using a measure of impurity, such as Gini impurity or entropy. The selected feature is used as the root node of the tree. Splitting: The dataset is split into subsets based on the possible values of the selected feature. Each subset corresponds to a branch of the tree, and the splitting process is repeated recursively for each subset. Stopping Criteria: The splitting process continues until a stopping criterion is met^6^. This could be reaching a maximum tree depth, having a minimum number of instances in a leaf node, or not achieving a significant improvement in impurity reduction. Leaf Node Assignment: Once the stopping criterion is met, the leaf nodes of the tree are assigned with the majority class label for classification tasks or the mean or median value for regression tasks. Prediction: To make predictions for new instances, the algorithm traverses the decision tree from the root node to a leaf node, following the splits based on the feature values. The prediction is based on the class label or target value associated with the reached leaf node. Decision trees have several advantages: They are easy to interpret and visualize, as the decision-making process is represented by a tree structure. Decision trees can handle both categorical and numerical features. They can capture nonlinear relationships between features. Decision trees can handle missing values and outliers in the data. However, there are considerations with decision trees: Decision trees are prone to overfitting, especially when the tree grows too deep or when the dataset contains noisy or irrelevant features. Techniques like pruning or using regularization parameters can help mitigate overfitting. Decision trees can be sensitive to small changes in the training data, which can lead to different tree structures and predictions. The algorithm may create complex and highly specific decision boundaries, which may not generalize well to unseen data. Decision trees may struggle with imbalanced datasets, as they can be biased towards the majority class. Despite these limitations, decision trees are widely used in various domains such as finance, healthcare, and customer relationship.

#### Random forest (RF)

**Figure 11:**
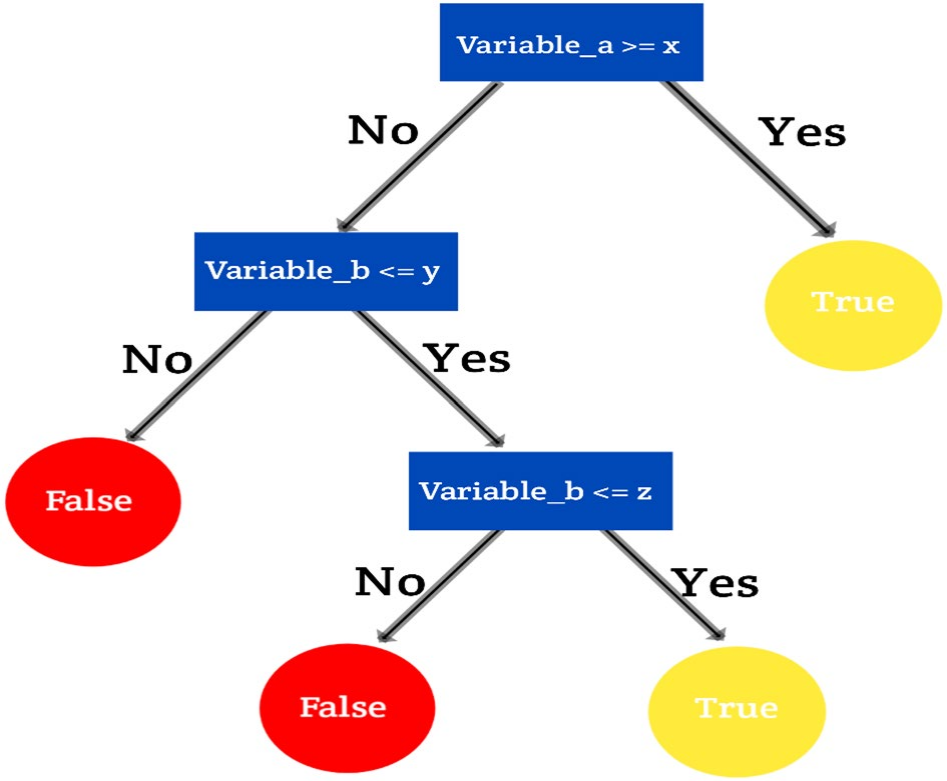
RF framework.

Random forest is an ensemble learning algorithm that combines multiple decision trees to create a more robust and accurate model^15^. The RF framework is presented in Fig. 11. It is a popular and powerful machine learning algorithm that is used for classification, regression, and other tasks. Here's an overview of how Random Forest works: Data Preparation: Prepare a labeled dataset where each instance consists of a set of features (attributes) and a corresponding class label or target value. Tree Construction: The Random Forest algorithm creates multiple decision trees using a subsample of the training data and a subset of the features. The subsample is typically generated by random sampling with replacement (i.e., bootstrapping), and the subset of features is randomly selected for each split. Tree Training: Each decision tree is trained on the subsample and the subset of features, using a similar process to the standard decision tree algorithm. Voting: To make predictions for new instances, the algorithm aggregates the predictions of all the decision trees. For classification tasks, the majority vote of the decision trees is taken as the predicted class label. For regression tasks, the mean or median value of the decision trees' predictions is taken as the predicted target value. Random Forest has several advantages: It is highly accurate and robust, as it combines the predictions of multiple decision trees, which reduces overfitting and improves generalization performance. Random Forest can handle nonlinear relationships between features. It can handle missing values and outliers in the data. Random Forest can be used for feature selection, as it provides an estimate of feature importance based on their contribution to the overall prediction. However, there are considerations with Random Forest: The algorithm can be computationally expensive, especially when dealing with large datasets or high-dimensional feature spaces. The interpretability of Random Forest is lower than that of a single decision tree, as the prediction process involves multiple decision trees. Random Forest may not perform as well as other algorithms, such as neural networks or gradient boosting, in certain scenarios. Overall, Random Forest is a powerful and versatile algorithm that has been successfully applied in various domains, including finance, healthcare, and natural language processing. It is implemented in popular machine learning libraries such as scikit-learn and TensorFlow.

### Symbolic regressions

#### Artificial neural network (ANN)

**Figure 12:**
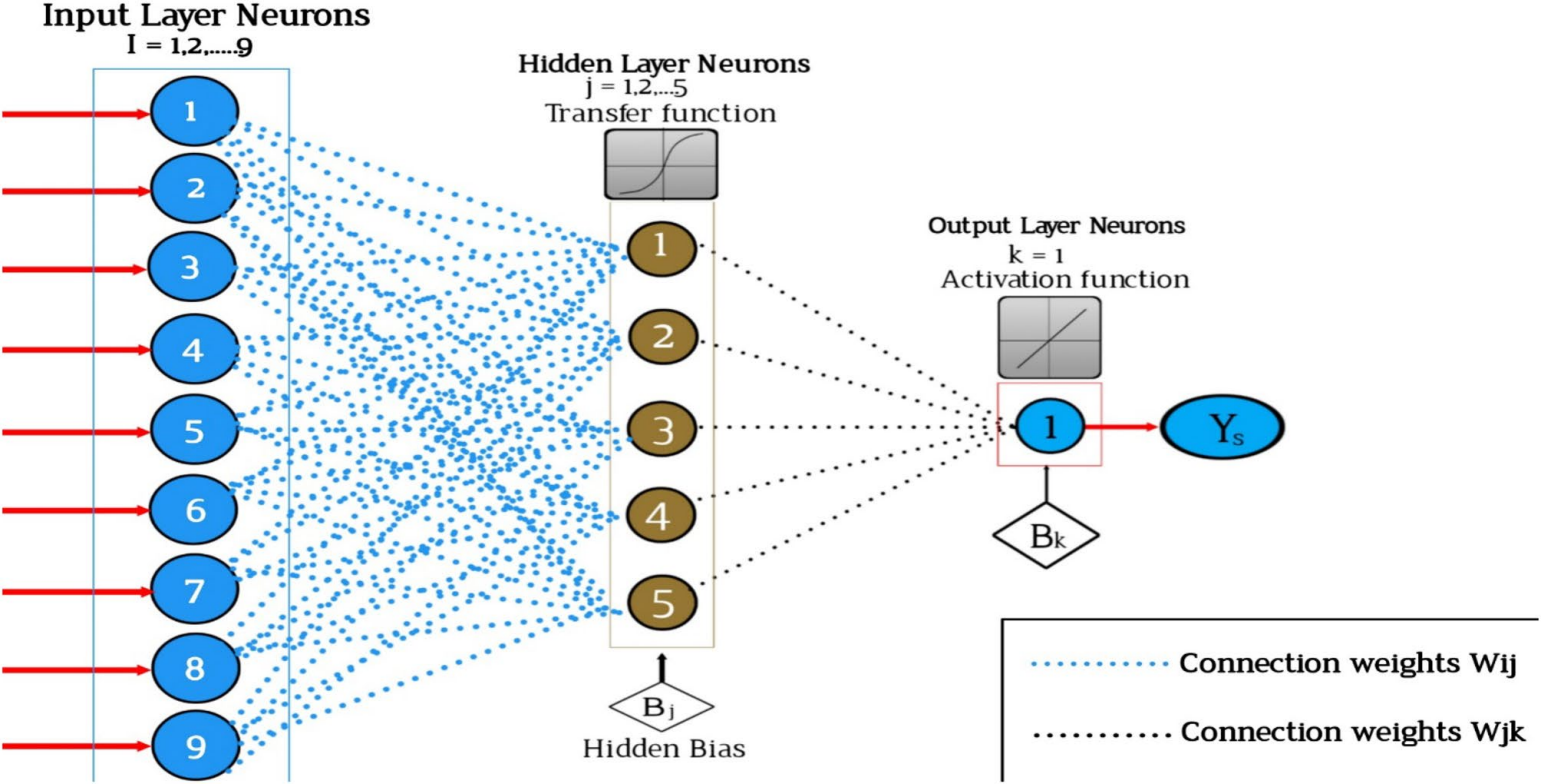
The framework of the ANN depicting activation function expression.

Artificial neural networks (ANNs) are computational models inspired by the structure and functionality of biological neural networks in the human brain^19^. The ANN framework is presented in Fig. 12. They are a subset of machine learning algorithms that have gained significant attention and success in various fields, including computer vision, natural language processing, speech recognition, and many others. An ANN consists of interconnected artificial nodes, called artificial neurons or simply "neurons". These neurons are organized into layers, typically divided into an input layer, one or more hidden layers, and an output layer. Each neuron receives input signals, processes them, and produces an output signal based on an activation function. The connections between neurons are represented by weights, which determine the strength and influence of one neuron on another. During the training process, these weights are adjusted iteratively based on a loss function, which measures the difference between predicted outputs and the desired outputs. This process is typically carried out using optimization algorithms like gradient descent, which aim to minimize the loss function. The most common type of ANN is the feedforward neural network, where signals flow only in one direction, from the input layer through the hidden layers to the output layer^19^. Another type is the recurrent neural network (RNN), which has connections that form loops, allowing information to persist and flow in cycles. RNNs are particularly suited for tasks involving sequential or temporal data, such as speech recognition or language translation. Deep neural networks (DNNs) are ANNs with multiple hidden layers. They have been particularly successful in recent years due to advances in computational power, availability of large datasets, and improvements in training algorithms. Deep learning, which refers to the use of DNNs, has achieved remarkable results in areas like image classification, object detection, text generation, and more. Overall, ANNs are powerful tools for pattern recognition, prediction, and decision-making tasks. They can automatically learn complex relationships and extract meaningful features from raw data, making them valuable tools in various domains. However, they also require careful design, training, and validation to ensure optimal performance and avoid overfitting or other issues.

#### Response surface methodology (RSM)

**Figure 13:**
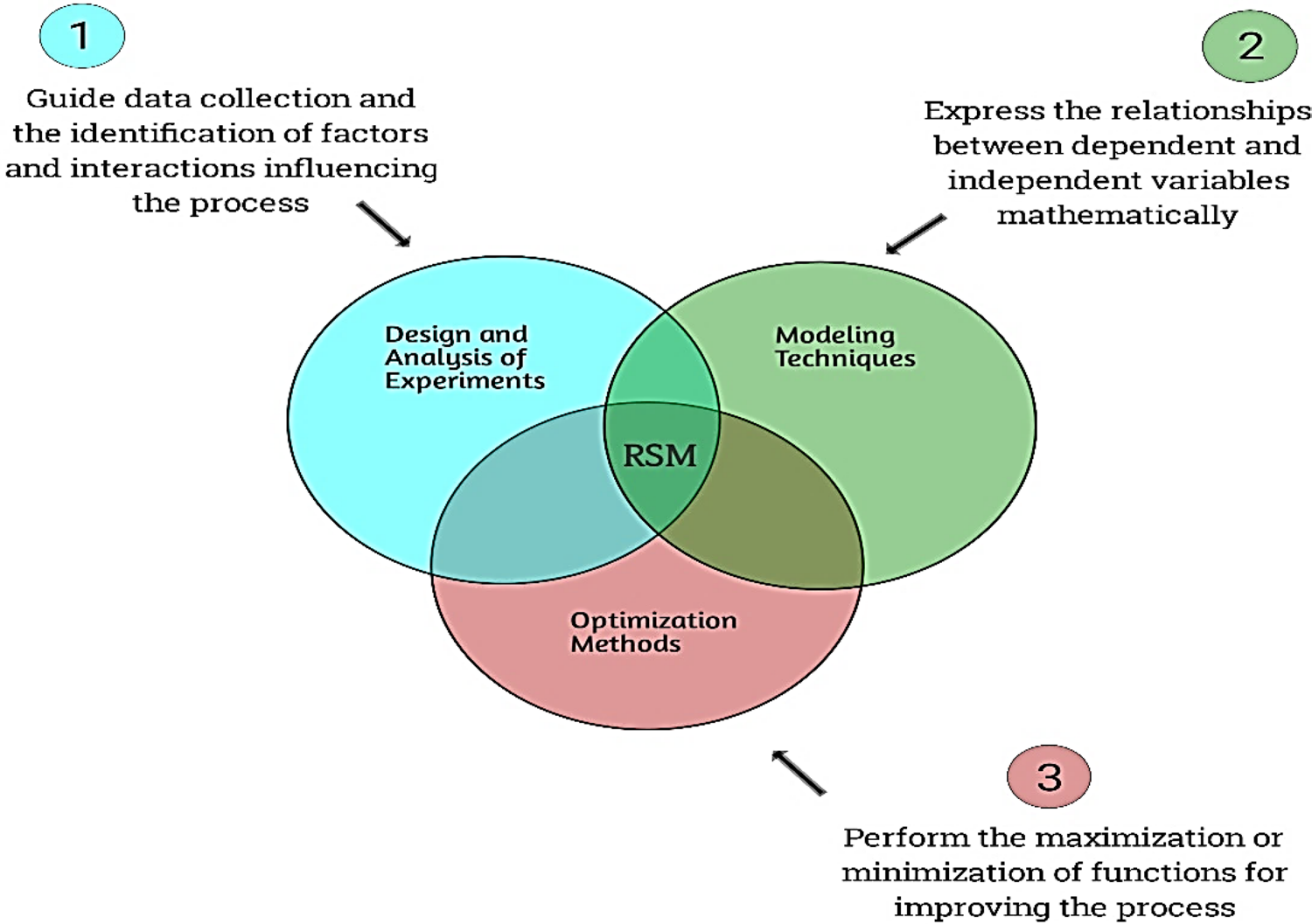
The operational framework of the RSM.

Response Surface Methodology (RSM) is a statistical and mathematical technique used for optimizing and modeling complex processes^21^. The RSM framework is presented in Fig. 13. It is particularly useful when the relationship between input variables and output responses is nonlinear and cannot be easily understood through simple linear models^21^. RSM is commonly employed in fields such as engineering, chemistry, manufacturing, and experimental design. The primary goal of RSM is to find the optimal settings of input variables that maximize or minimize a response of interest^22^. The process involves conducting a series of experiments or simulations to collect data on the response variable at different combinations of input variables^23^. These data points are then used to fit a mathematical model that describes the relationship between the input variables and the response^24^. The model is typically constructed using regression techniques such as polynomial regression. The response surface, as the name suggests, is a visual representation of the relationship between the input variables and the response^25^. It is a three-dimensional plot where the axes represent the input variables, and the surface represents the response. By analyzing the response surface, researchers can identify the optimal values of the input variables that lead to the desired response^26^. RSM allows for the optimization of processes by finding the combination of input variables that maximizes a desired output response or minimizes undesired responses. It helps in reducing the number of experiments needed to determine the optimal settings and provides insights into the interactions between variables^27^. RSM can also be used for sensitivity analysis to identify the most critical factors affecting the response. In summary, Response Surface Methodology is a powerful statistical technique used for optimizing complex processes by modeling the relationship between input variables and output responses. It helps researchers and engineers make informed decisions and improve the efficiency and effectiveness of their processes.

### Sensitivity analysis

In the context of modeling and analysis, sensitivity analysis refers to the study of how the uncertainty in the output of a model or system can be attributed to different sources of uncertainty in its input variables^28^. Sensitivity analysis helps identify which input variables have the most significant impact on the output and how changes in input variables affect the output of interest^29^. There are various methods for conducting sensitivity analysis, including: One-at-a-Time (OAT) Sensitivity Analysis: This method involves varying one input variable at a time while keeping all other variables constant at their base values^30^. It helps assess the individual effect of each input variable on the output but does not capture potential interactions between variables. Local Sensitivity Analysis: Local sensitivity analysis calculates the sensitivity of the output to changes in the input variables around a specific point or scenario^31^. It provides insights into how small perturbations in input variables near the current operating point affect the output. Global Sensitivity Analysis (GSA): GSA considers the entire input parameter space and evaluates the sensitivity of the output to variations in input variables across the entire range^31^. It helps identify the most influential input variables and interactions between variables, even in high-dimensional and nonlinear systems. Common GSA methods include: Variance-Based Sensitivity Analysis (e.g., Sobol' indices): Decomposes the variance of the output into contributions from individual input variables or combinations of variables. Screening Methods (e.g., Morris method): Identifies influential input variables using a limited number of model evaluations. Response Surface-Based Methods (e.g., polynomial chaos expansion): Approximates the model response using a surrogate model and evaluates the sensitivity of the surrogate model to input variations^32^. Graphical Methods: Graphical techniques such as tornado diagrams, scatter plots, and sensitivity curves provide visual representations of the sensitivity of the output to changes in input variables. They offer intuitive insights into the relative importance of input variables and their relationships^33^. Monte Carlo Simulation: Monte Carlo simulation involves sampling input variables from their probability distributions and propagating them through the model to estimate the distribution of the output^34^. Sensitivity analysis can be performed by examining how changes in input distributions affect the output distribution. Sensitivity analysis is crucial for decision-making, risk assessment, and model validation in various fields such as engineering, finance, environmental science, and healthcare. It helps stakeholders understand the uncertainties associated with model predictions and identify key drivers of variability in the system under study^35^. A preliminary sensitivity analysis was carried out on the collected database to estimate the impact of each input on the (Fc) values. “Single variable per time” technique is used to determine the “Sensitivity Index” (SI) for each input using Hoffman and Gardener formula^35^ as follows:

1 $$SI \left({X}_{n}\right)= \frac{Y\left({X}_{max}\right)-Y\left({X}_{min}\right)}{Y\left({X}_{max}\right)}$$

### Performance evaluation indices

The following section discusses the results of each model. The accuracies of developed models were evaluated by comparing SSE, MAE, MSE, RMSE, Error %, Accuracy % and R^2^ between predicted and calculated shear strength parameters values. The definition of each used measurement is presented in Eqs. (2)–(7)^37^.

2 $$MAE= \frac{1}{N}\sum_{i=1}^{N}\left|{y}_{i}-\widehat{y}\right|$$

3 $$MSE= \frac{1}{N}\sum_{i=1}^{N}{\left({y}_{i}-\widehat{y}\right)}^{2}$$

4 $$RMSE= \sqrt{MSE}$$

5 $$Error \%=\frac{RMSE}{\widehat{y}}$$

6 $$Accurcy \%=1-Error \%$$

7 $${R}^{2}=1- \frac{\sum {\left({y}_{i}-\widehat{y}\right)}^{2}}{\sum {\left({y}_{i}-\overline{y }\right)}^{2}}$$

where N is the total number of entries, ${y}_{i}$ is the ith entries, $\widehat{y}$ is the modal value, $\overline{y }$ is the average value.

### Consent to participate

The authors have the consent to participate in this publication of the research paper.

## Results presentation and discussion

### GB model

**Figure 14:**
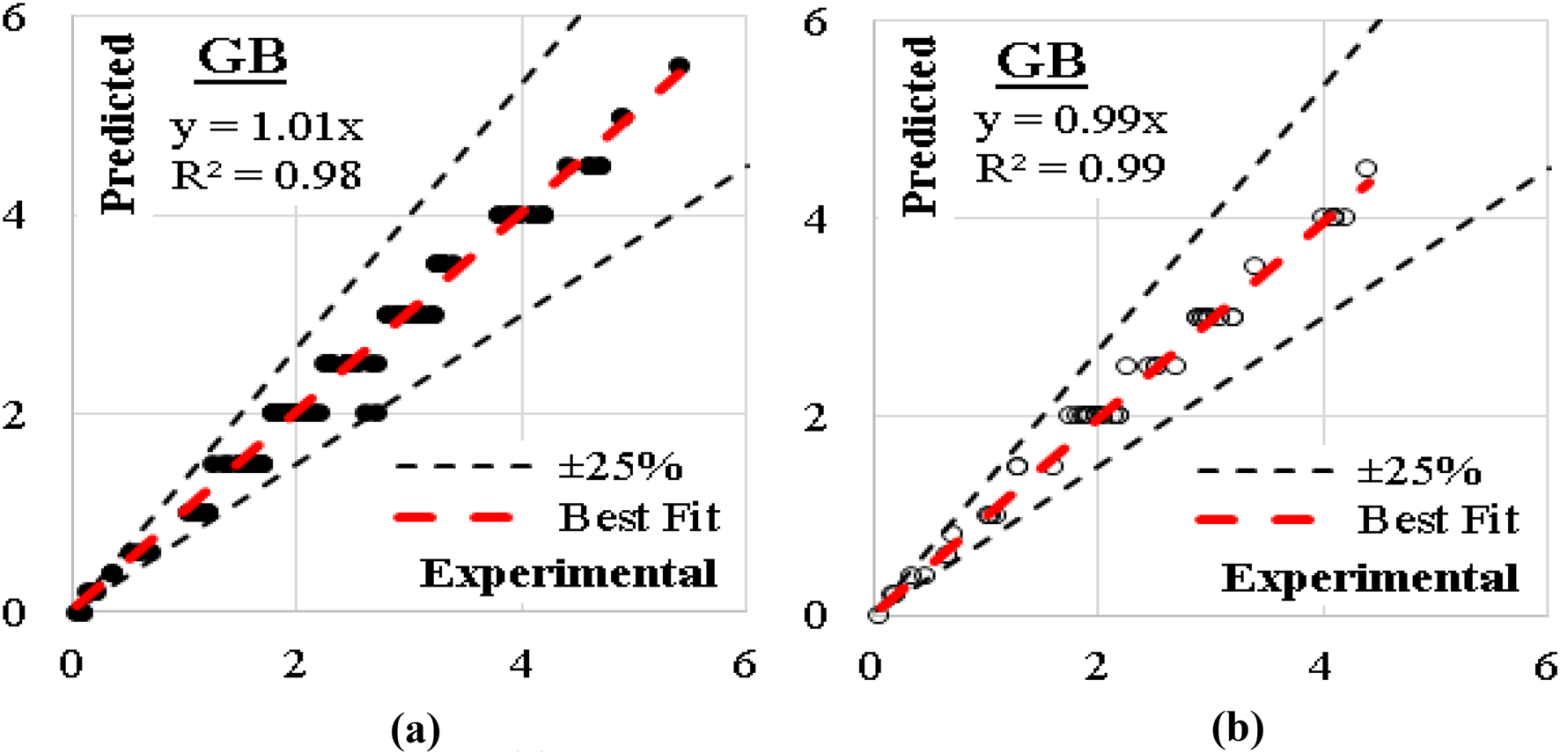
Relation between predicted and calculated (UCS) values using (GB) (**a**) Training set, (**b**) Validation set.

The developed (GB) model was based on (Scikit-learn) method with maximum number of trees of 100 and learning rate of 0.1. The growth control was limited by maximum number of tree levels of 9 and minimum splitting subset of 2. Finally, the fraction of training instances was conspired 1.0. The errors % and (R^2^) values for this model were (6%, 0.98) for training dataset and (5%, 0.99) for validation dataset. The relations between calculated and predicted values are shown in Fig. 14.

### CN2 model

**Figure 15:**
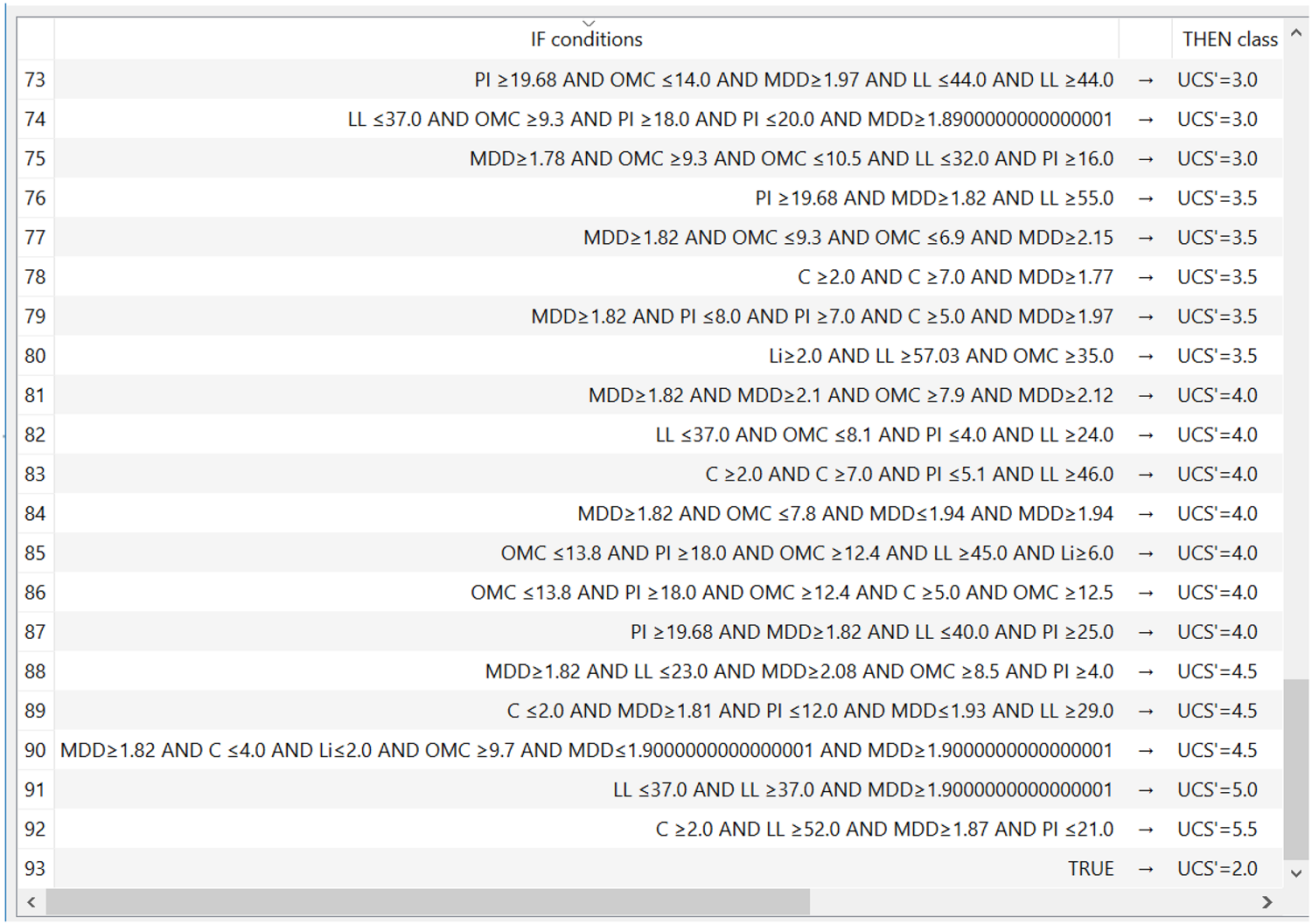
Part of the (IF-condition) rules of the developed (CN2) model.

**Figure 16:**
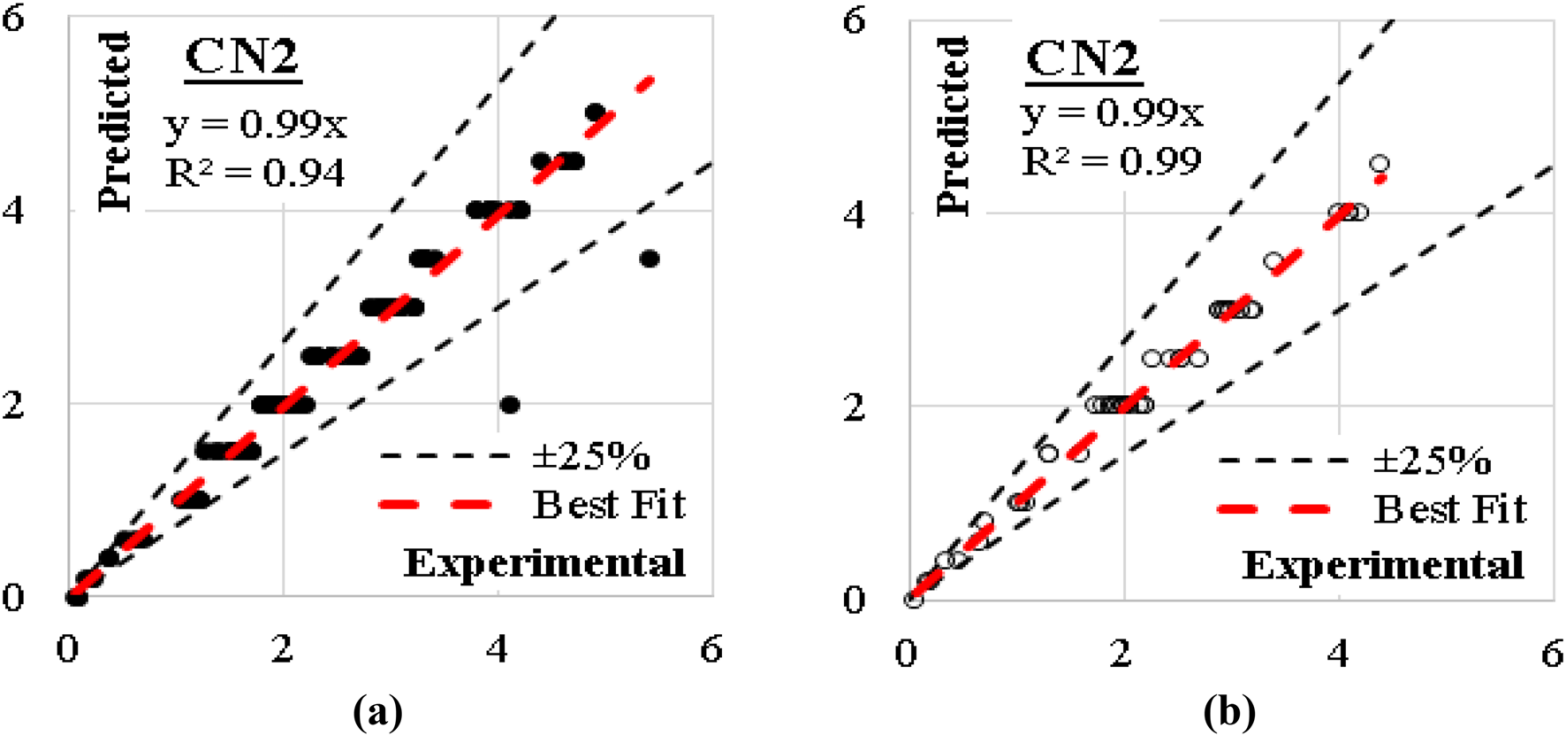
Relation between predicted and calculated (UCS) values using (CN2) (**a**) Training set, (**b**) Validation set.

The developed (CN2) model was created considering “Laplace accuracy” as evaluation measurement with beam width of 6.0. The rule filtering criteria were limited to maximum rule length of 6.0 and minimum rule coverage of 1.0. The generated model had 93 “IF condition” rules, Fig. 15 presents some of them. The errors % and (R^2^) values for this model were (11%, 0.94) for training dataset and (5%, 0.99) for validation dataset. The relations between calculated and predicted values are shown in Fig. 16.

### NB model

**Figure 17:**
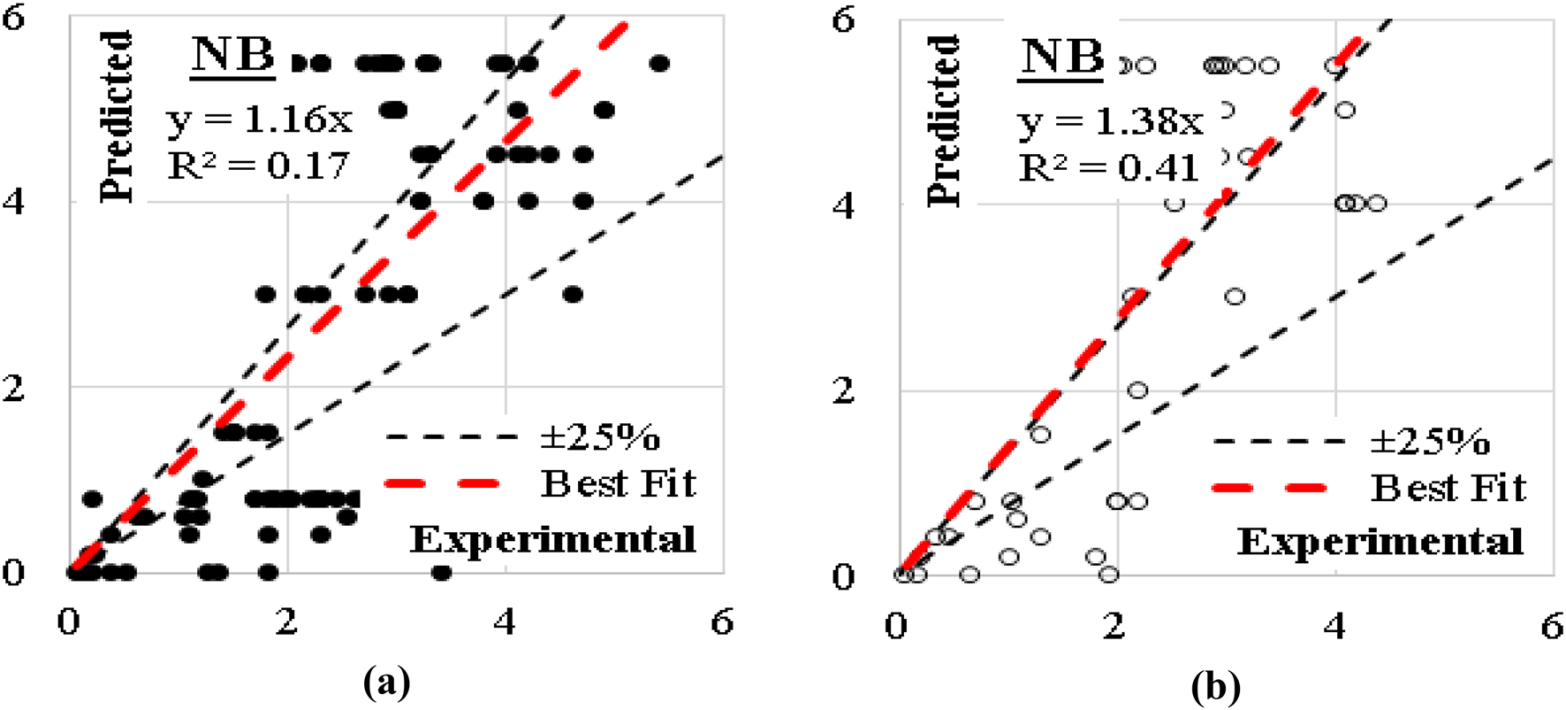
Relation between predicted and calculated (UCS) values using (NB) (**a**) Training set, (**b**) Validation set.

Traditional Naive Bayes classifier technique considering the concept of “Maximum likelihood” were used to develop this model. Although this type of classifier is highly scalable and are used in many applications, but it showed a very low performance where the errors % and (R^2^) values were (87%, 0.17) for training dataset and (86%, 0.41) for validation dataset. The relations between calculated and predicted values are shown in Fig. 17.

### SVM model

**Figure 18:**
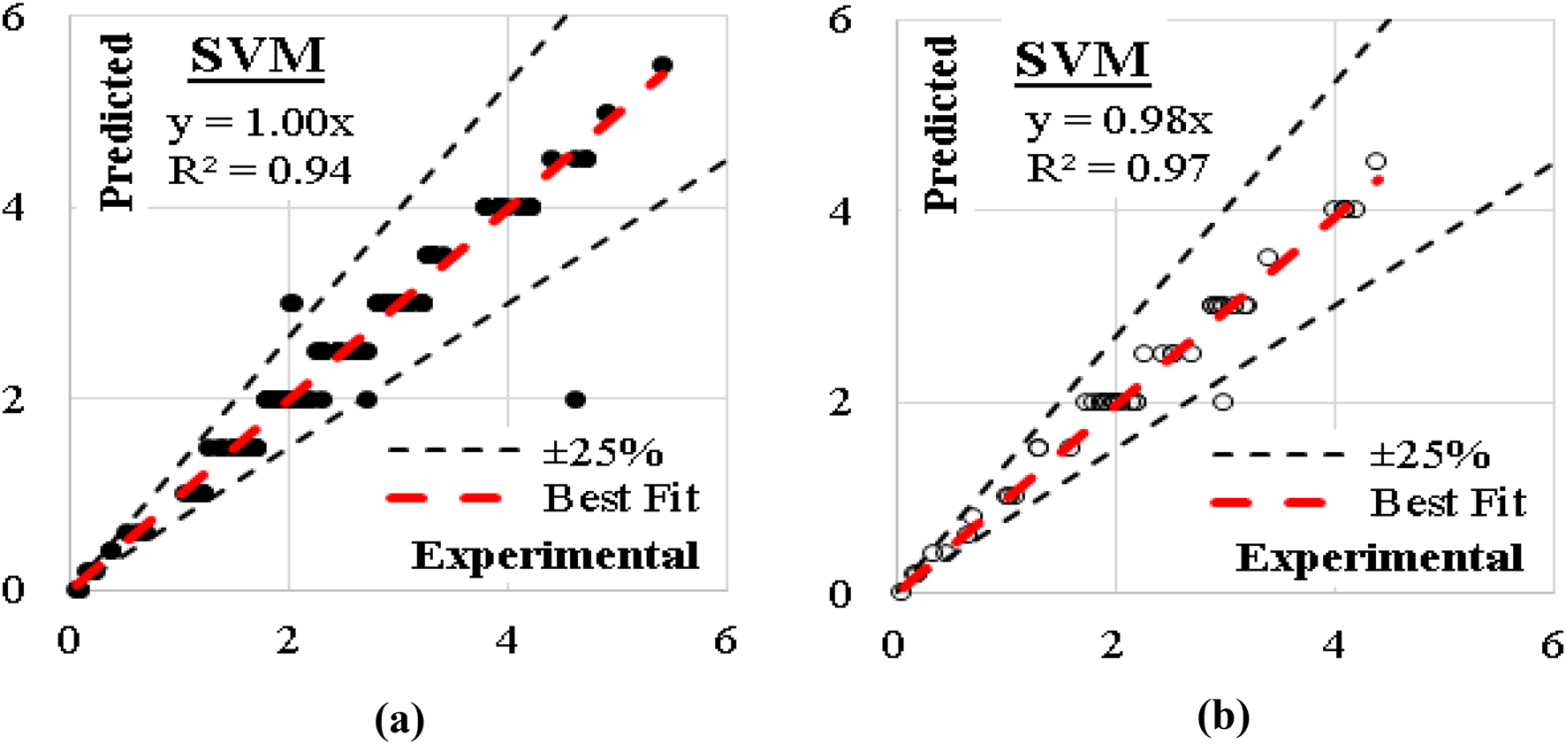
Relation between predicted and calculated (UCS) values using (SVM) (**a**) Training set, (**b**) Validation set.

The developed (SVM) model was based on “Quadrilateral polynomial” kernel with cost value of 100, regression loss of 0.10 and numerical tolerance of 1.0. These configurations were enough of generate a model with errors % and (R^2^) values of (12%, 0.94) for training dataset and (6%, 0.97) for validation dataset. The relations between calculated and predicted values are shown in Fig. 18.

### SGD model

**Figure 19:**
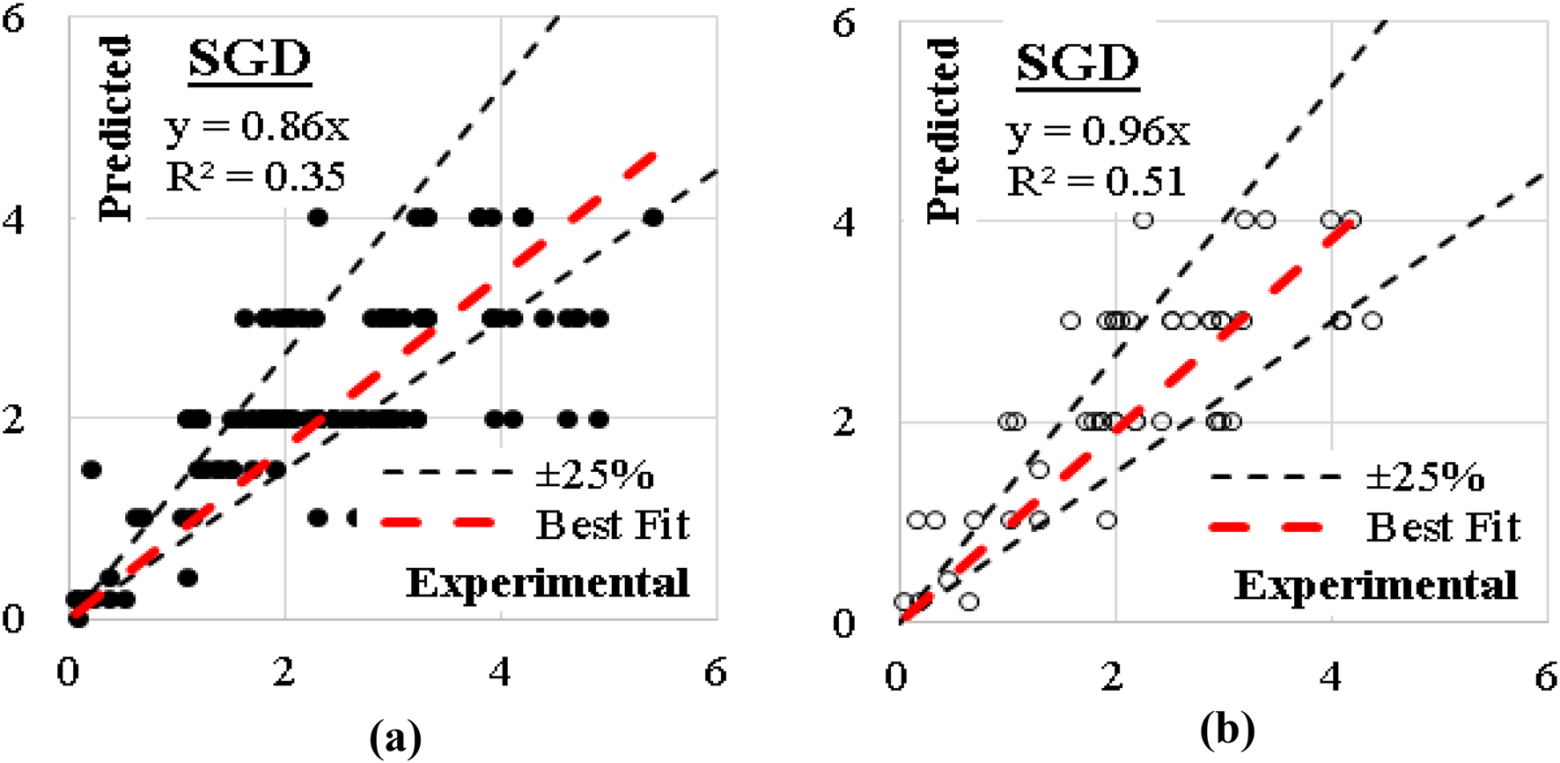
Relation between predicted and calculated (UCS) values using (SGD) (**a**) Training set, (**b**) Validation set.

This model was developed considering modified Huber classification function and “Elastic net” re-generalization technique with mixing factor of 0.01 and strength factor of 0.001. The errors % of and (R^2^) values were (35%, 0.35) for training dataset and (33%, 0.51) for validation dataset. The relations between calculated and predicted values are shown in Fig. 19.

### K-NN model

**Figure 20:**
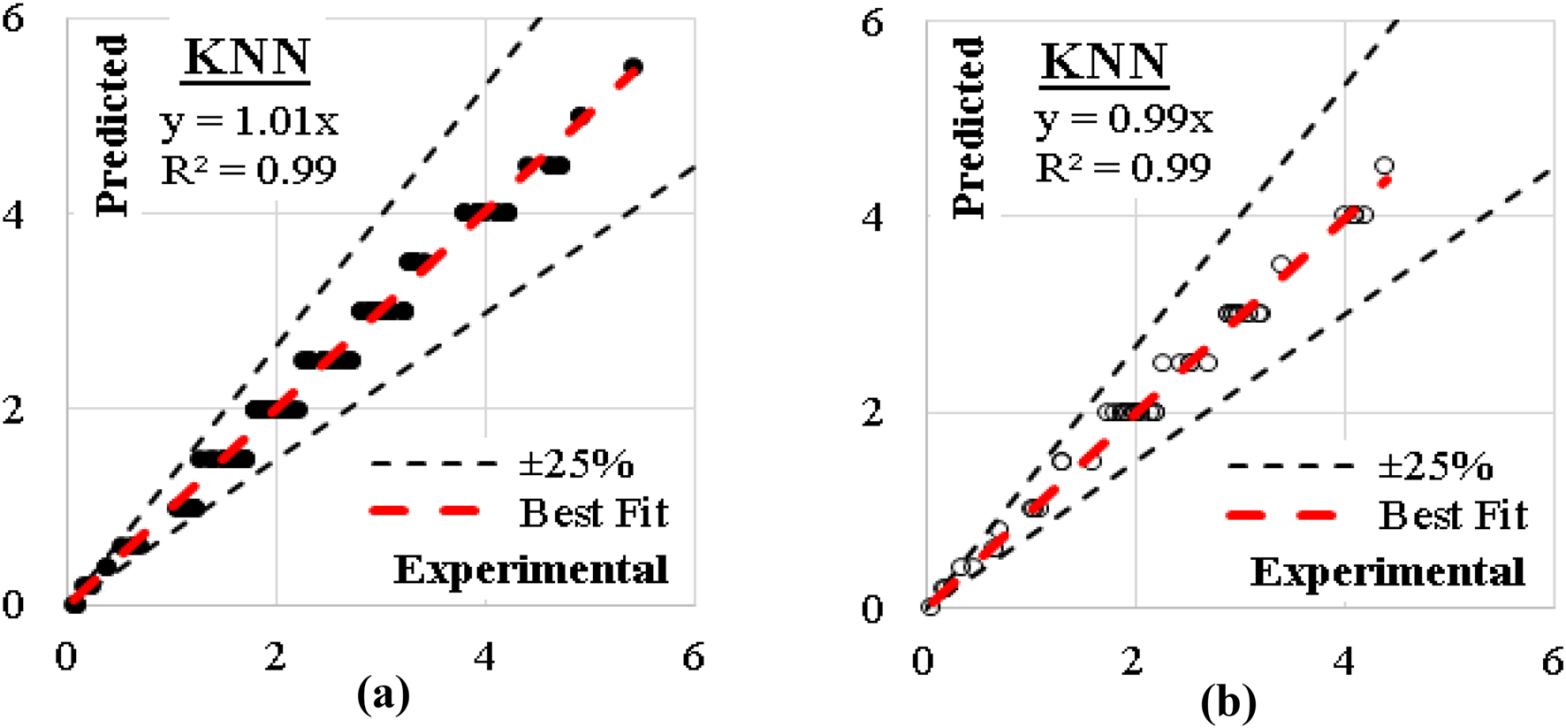
Relation between predicted and calculated (UCS) values using (KNN) (**a**) Training set, (**b**) Validation set.

Considering number of neighbors of 1.0, Euclidian metric method and weights were evaluated by distances, the developed (K-NN) models showed the best accuracy. The errors % of and (R^2^) values were (6%, 0.99) for training dataset and (5%, 0.99) for validation dataset. The relations between calculated and predicted values are shown in Fig. 20.

### Tree model

**Figure 21:**
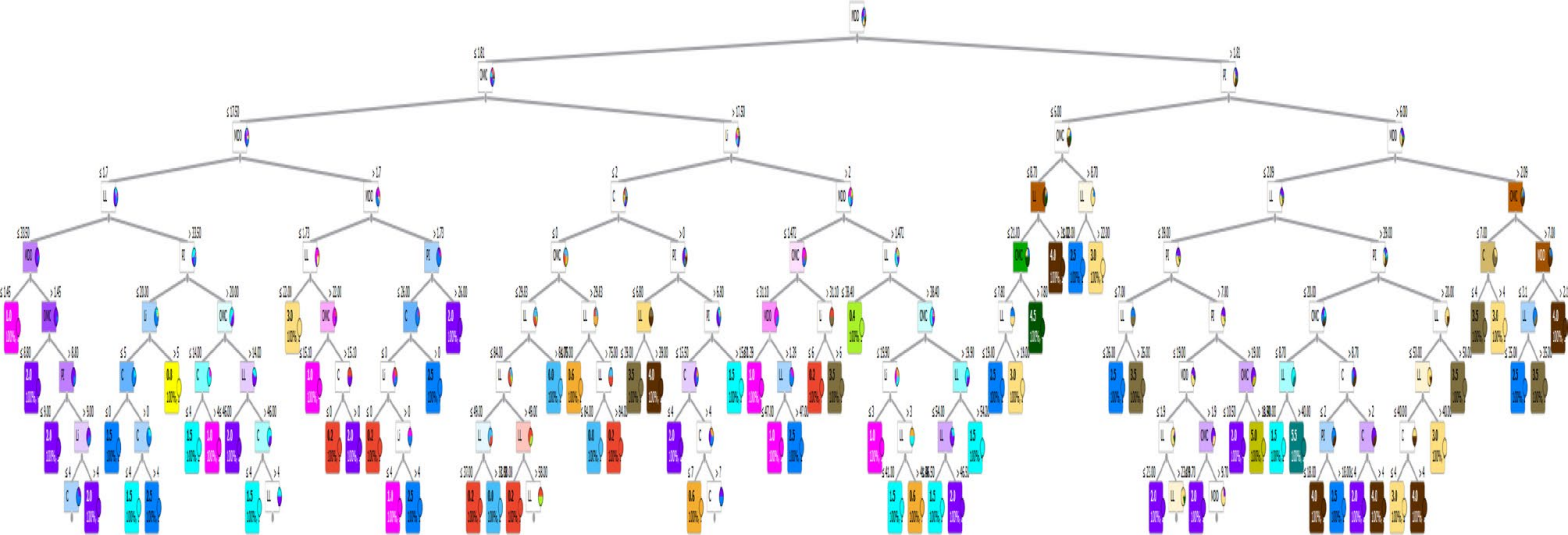
The layout of the developed (Tree) model.

**Figure 22:**
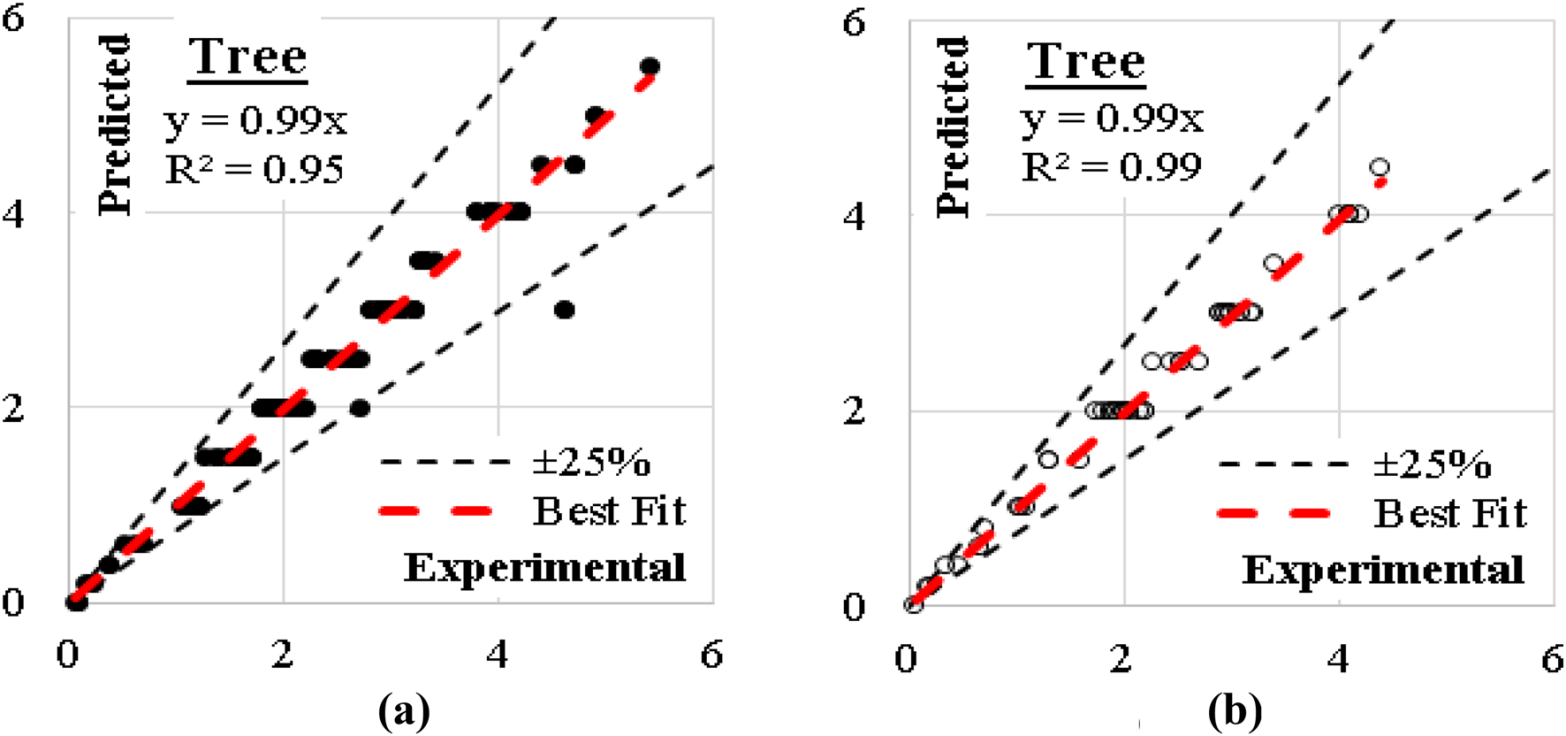
Relation between predicted and calculated (UCS) values using (Tree) (**a**) Training set, (**b**) Validation set.

This model was developed considering maximum number of tree levels of 9.0, minimum number of instants in leaves of 2.0 and minimum split subset of 5.0. The layout of the generated model is presented in Fig. 21. The developed model showed an excellent accuracy with errors % and (R^2^) values were (9%, 0.95) for training dataset and (5%, 0.99) for validation dataset. The relations between calculated and predicted values are shown in Fig. 22.

### RF model

**Figure 23:**
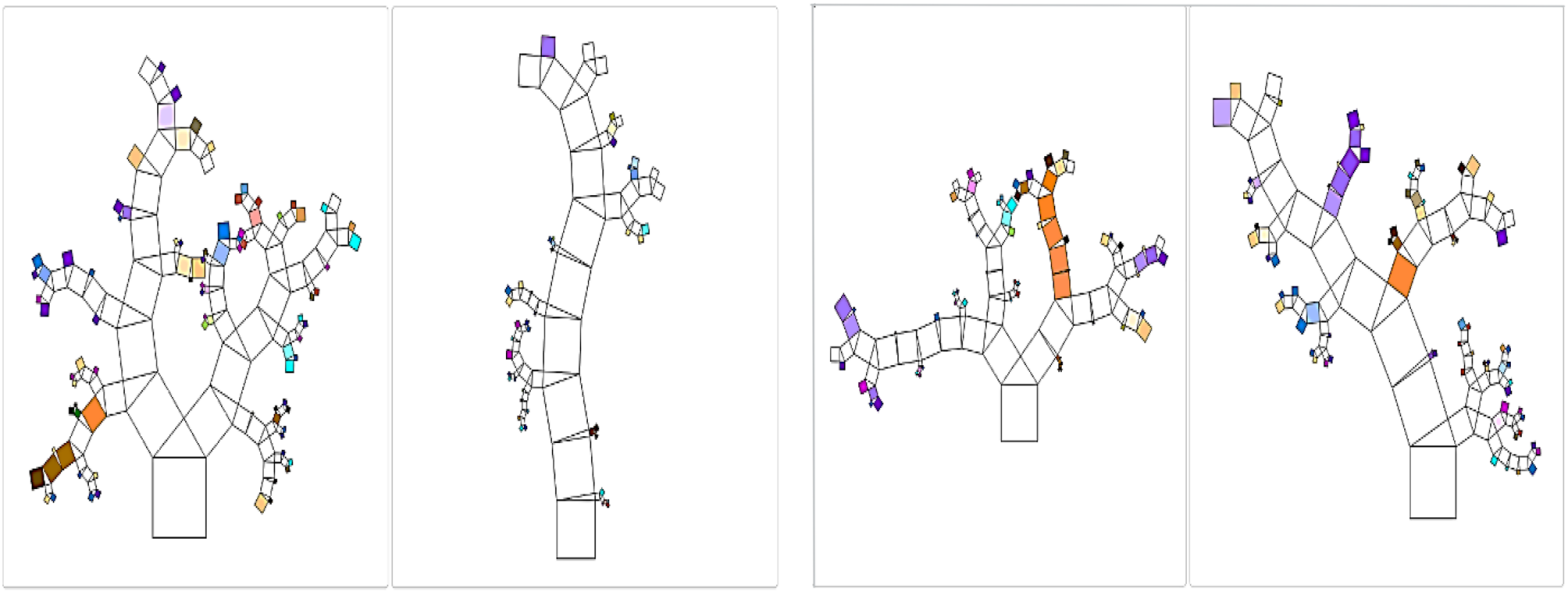
Pythagorean Forest diagram for the developed (RF) model.

**Figure 24:**
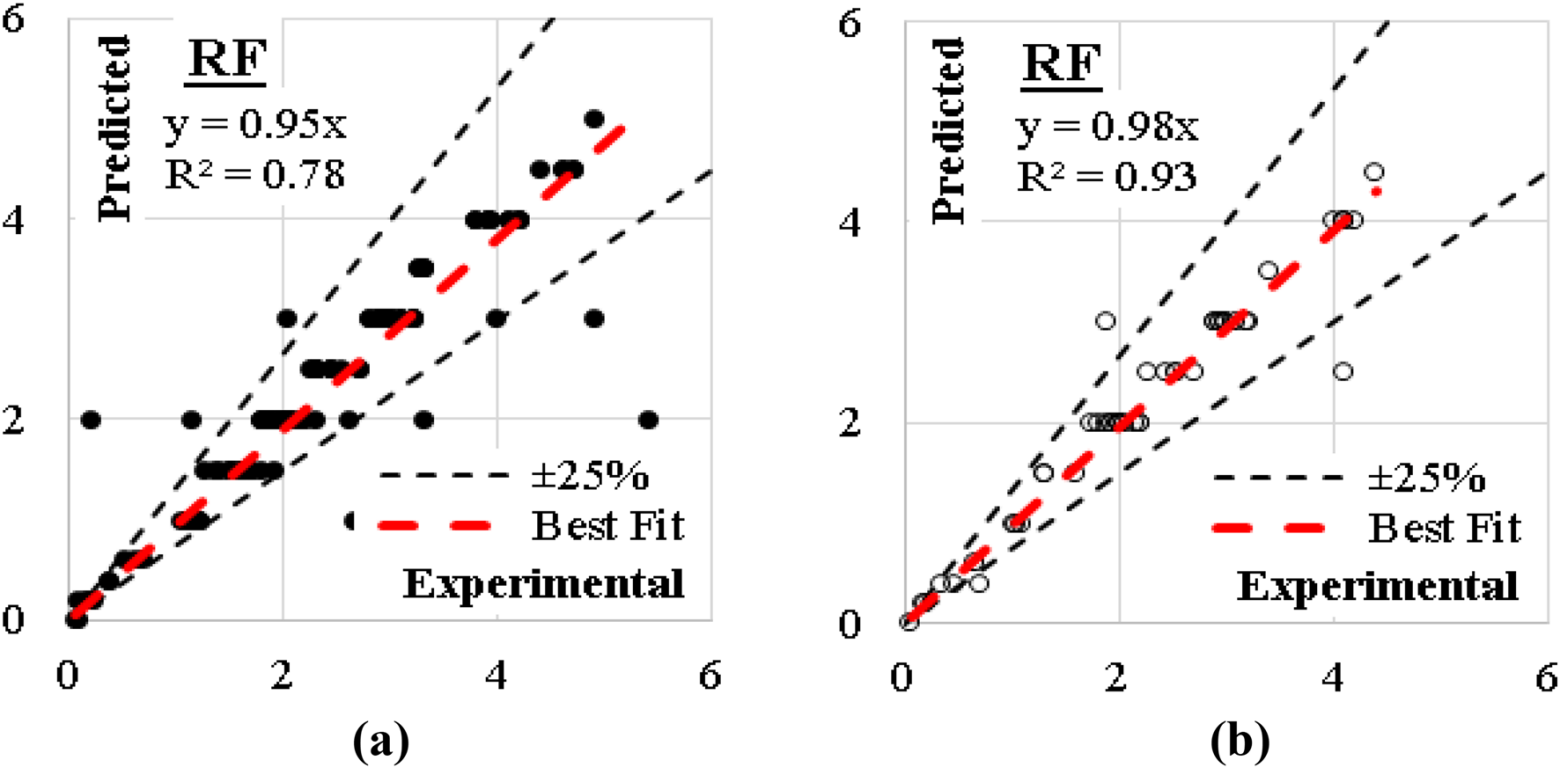
Relation between predicted and calculated (UCS) values using (RF) (**a**) Training set, (**b**) Validation set.

Finally, the generated (RF) model was based on only four trees with number of levels limited to 9.0. The developed model is graphically presented using Pythagorean Forest in Fig. 23. This arrangement leaded to a fair accuracy where errors % and (R^2^) values were (21%, 0.78) for training dataset and (13%, 0.93) for validation dataset. The relations between calculated and predicted values are shown in Fig. 24.

**Figure 25:**
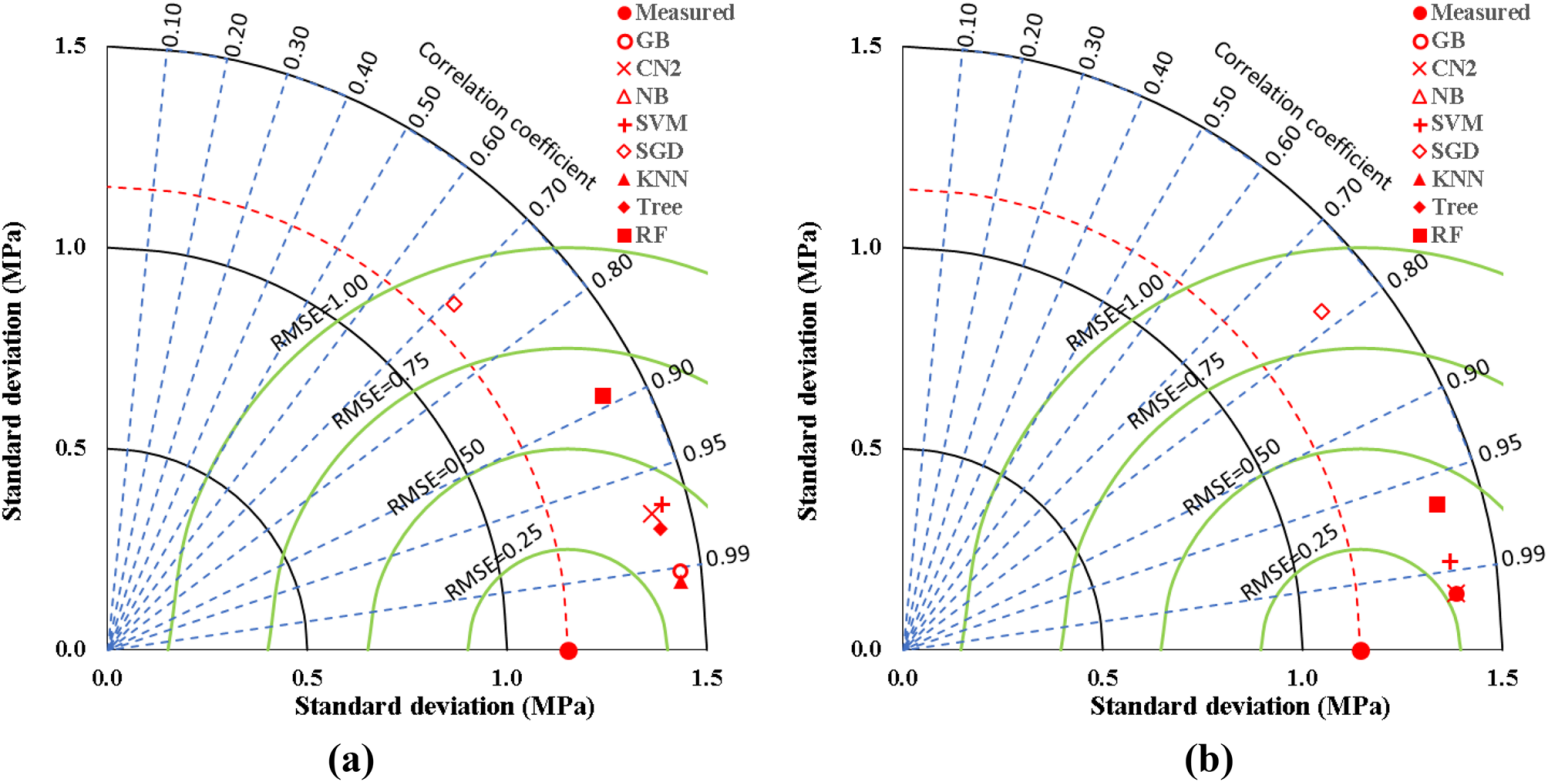
Comparing the accuracies of the developed models using Taylor charts, (**a**) Training dataset, (**b**) Validation dataset.

The ensemble-based models on overall have shown remarkable abilities in the prediction of the UCS of cement-lime reconstituted cohesive soil. The summary of the performance evaluation analysis has presented in Table 3. Figure 25 represents plots comparing the accuracies of the developed models using Taylor charts. It can be shown that the K-NN produced the best model, followed by GB model and Tree model in that order. Conversely, the NB performed very poorly compared to the other techniques in the ensemble group. The best performed models can be applied in the design of optimal behavior of reconstituted soils for the purpose of foundation constructions.

**Table 3:** Performance measurements of developed ensemble models.

Model	Dataset	SSE	MAE	MSE	RMSE	Error	Accuracy	R^2^
		–	kN/m^2^	(kN/m^2^)^2^	kN/m^2^	%	%	–
GB	Training	4.23	0.12	0.02	0.15	0.06	0.94	0.98
	Validation	0.68	0.09	0.01	0.12	0.05	0.95	0.99
CN2	Training	11.41	0.13	0.06	0.25	0.11	0.89	0.94
	Validation	0.68	0.09	0.01	0.12	0.05	0.95	0.99
NB	Training	771.86	1.60	4.06	2.02	0.87	0.13	0.17
	Validation	186.07	1.51	3.72	1.93	0.86	0.14	0.41
SVM	Training	13.62	0.14	0.07	0.27	0.12	0.88	0.94
	Validation	1.68	0.11	0.03	0.18	0.06	0.94	0.97
SGD	Training	123.27	0.59	0.65	0.81	0.35	0.65	0.35
	Validation	26.68	0.57	0.03	0.73	0.33	0.67	0.51
KNN	Training	3.41	0.11	0.02	0.13	0.06	0.94	0.99
	Validation	0.68	0.09	0.01	0.12	0.05	0.95	0.99
Tree	Training	8.96	0.13	0.05	0.22	0.09	0.91	0.95
	Validation	0.68	0.09	0.01	0.12	0.05	0.95	0.99
RF	Training	43.54	0.21	0.23	0.48	0.21	0.79	0.78
	Validation	4.56	0.15	0.09	0.30	0.13	0.87	0.93

### ANN and RSM comparative suitability check models

#### ANN

**Figure 26:**
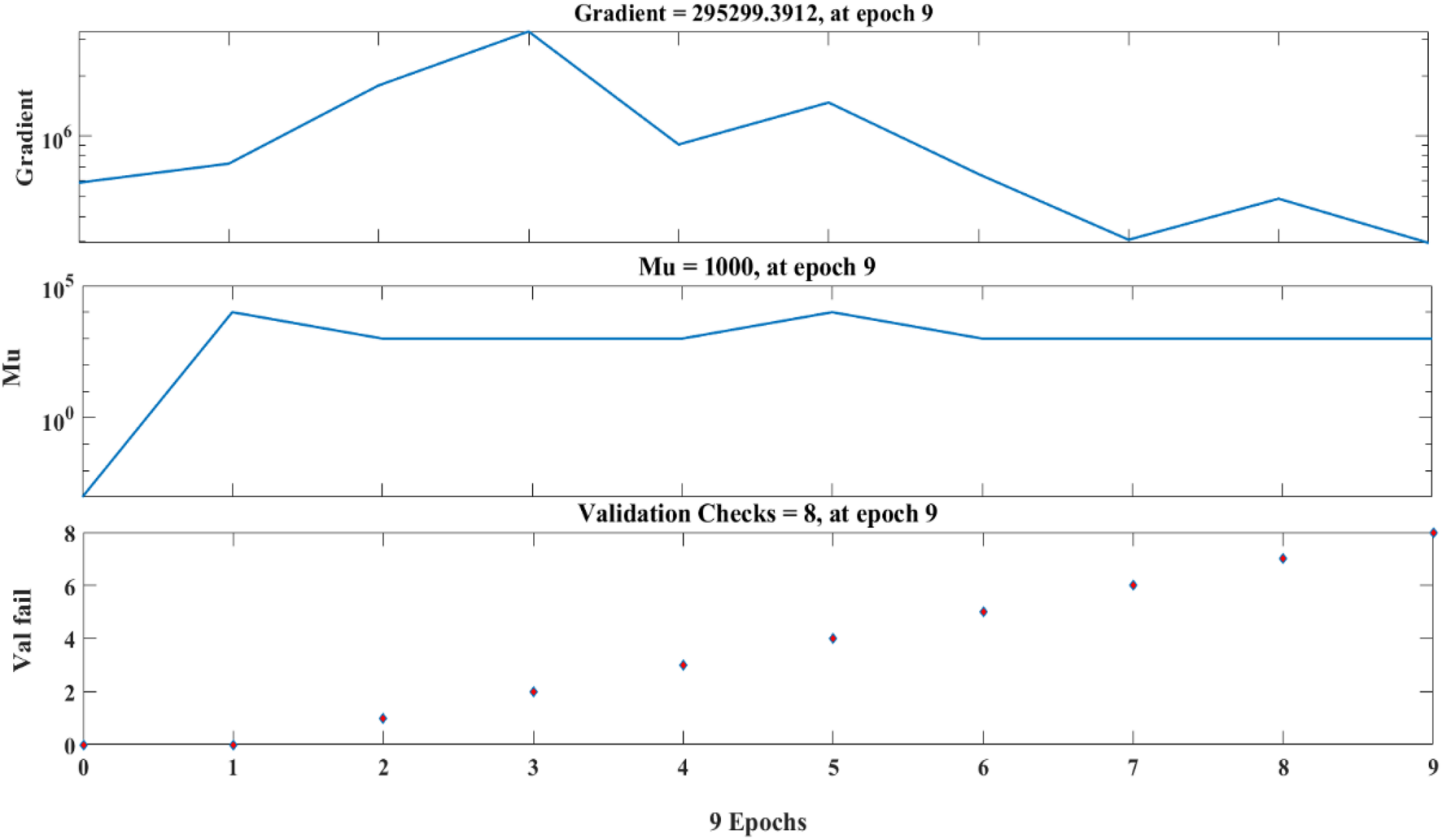
The ANN epochs.

The epoch of the ANN analysis is presented in Fig. 26. Analyzing the results of an Artificial Neural Network (ANN) training, particularly in terms of epochs, involves understanding how the performance of the network changes over the course of training iterations as presented in Fig. 26.

**Figure 27:**
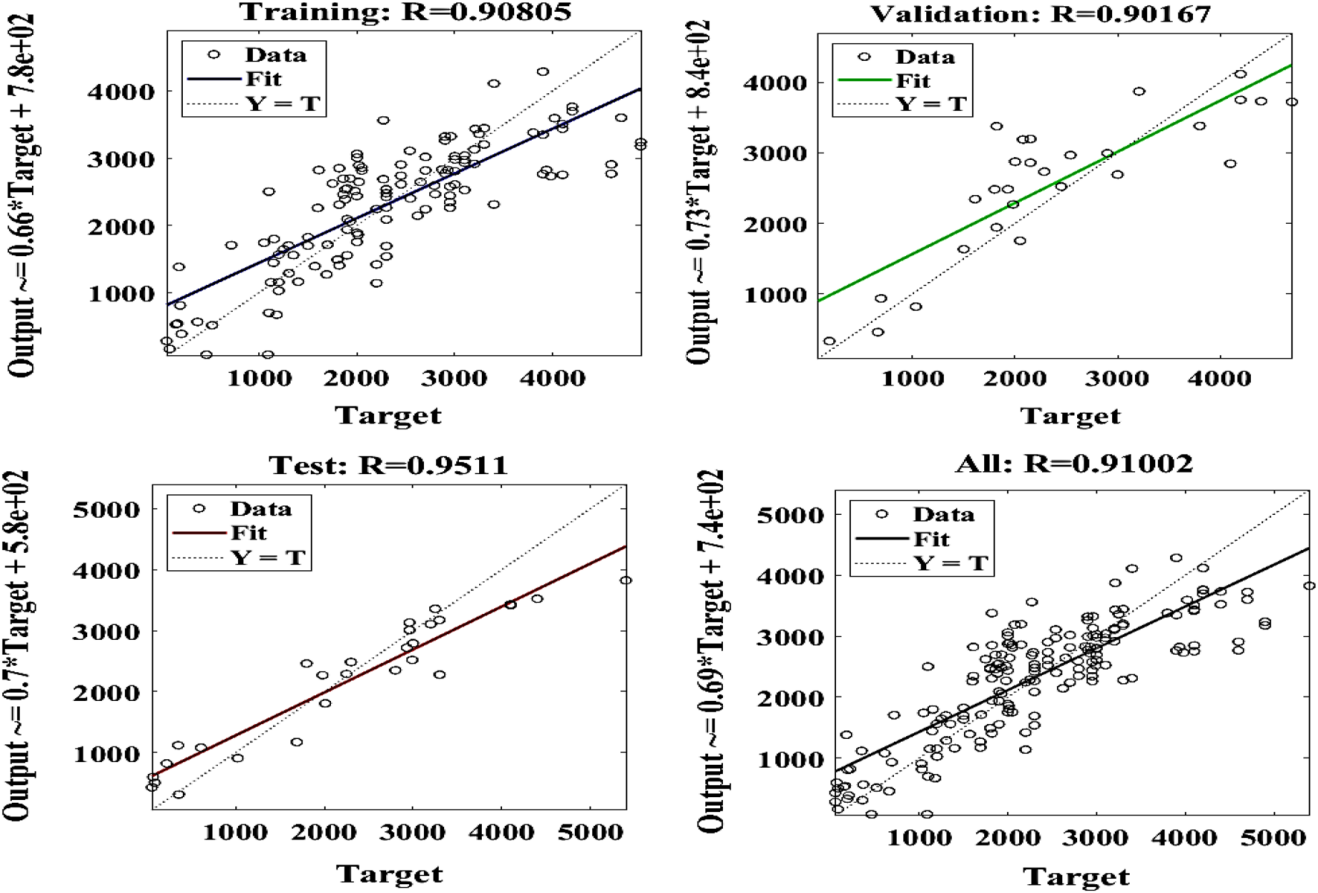
The ANN model outcome.

**Figure 28:**
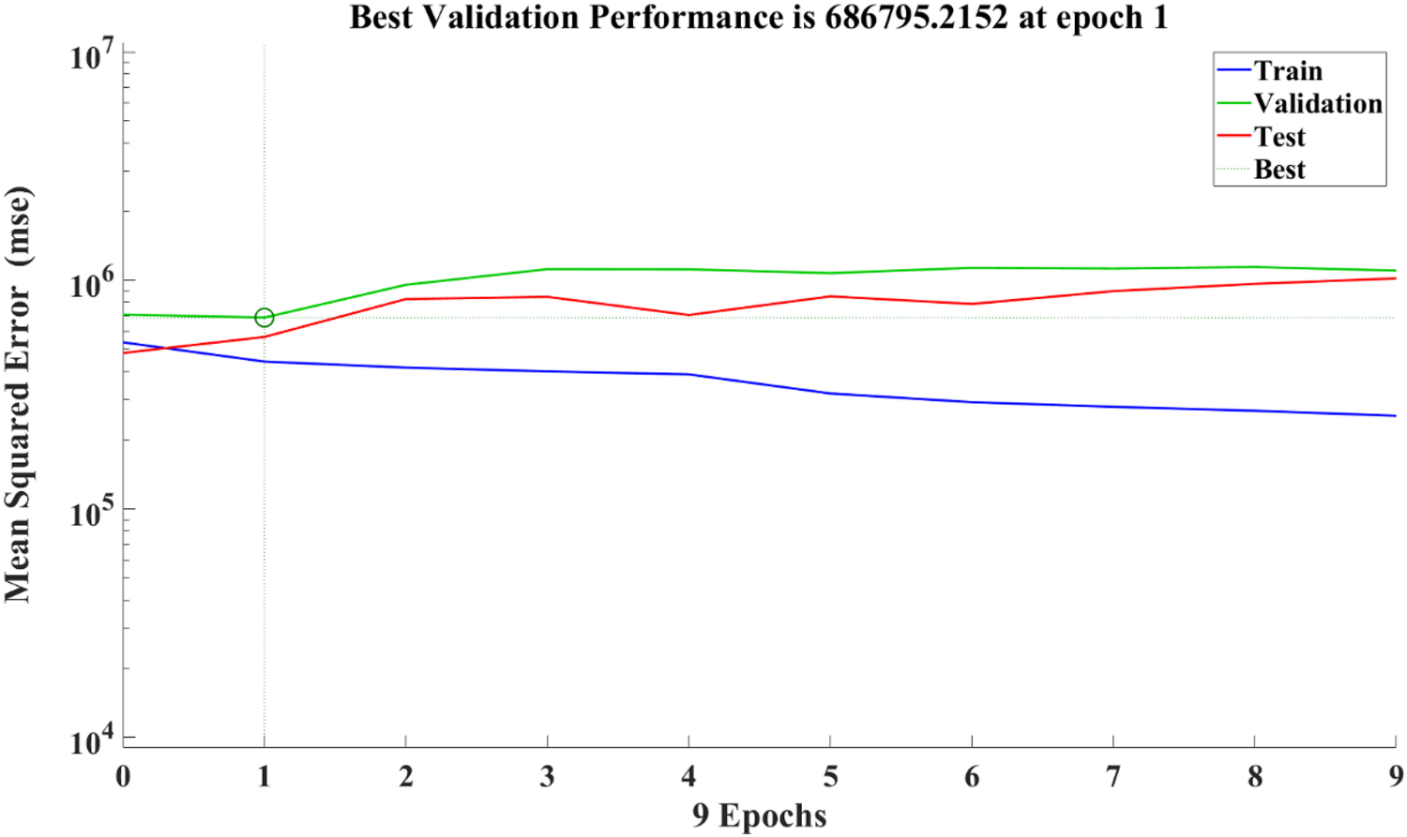
The best validation performance.

Typically, the loss decrease as the network learns from the training data. However, signs of overfitting are monitored, where the loss starts to increase on the validation set while continuing to decrease on the training set. The learning curve shows the evolution of the performance metric on both the training and validation sets over epochs. This curve provides insights into whether the model is learning effectively from the data and whether it is generalizing well to unseen data. The performance of the model on a validation set was monitored also and implementing early stopping was considered. Early stopping involves halting the training process when the performance on the validation set stops improving or starts to deteriorate. This helped to prevent overfitting and ensures that the model is not trained for too many epochs, which can lead to poor generalization. The weights of the network's connections and the activations of neurons in different layers over epochs were visualized. This helped identify patterns in how the network is learning and whether there are any issues such as vanishing gradients or dead neurons. The changes in hyperparameters (e.g., learning rate, batch size, network architecture) that may have affected the network's performance over epochs were analyzed. By analyzing the ANN epoch results using these approaches, can help a research gain insights into how the network is learning and improving over time, identify potential issues, and make informed decisions about model training and hyperparameter tuning. The fit plots for that compared the actual and predicted values are presented in Fig. 27. The ANN produced an average R value of 0.91 to compare closely with the ensemble-based models. The validation of the best performance is presented in Fig. 28

#### Response surface methodology (RSM) model

**Figure 29:**
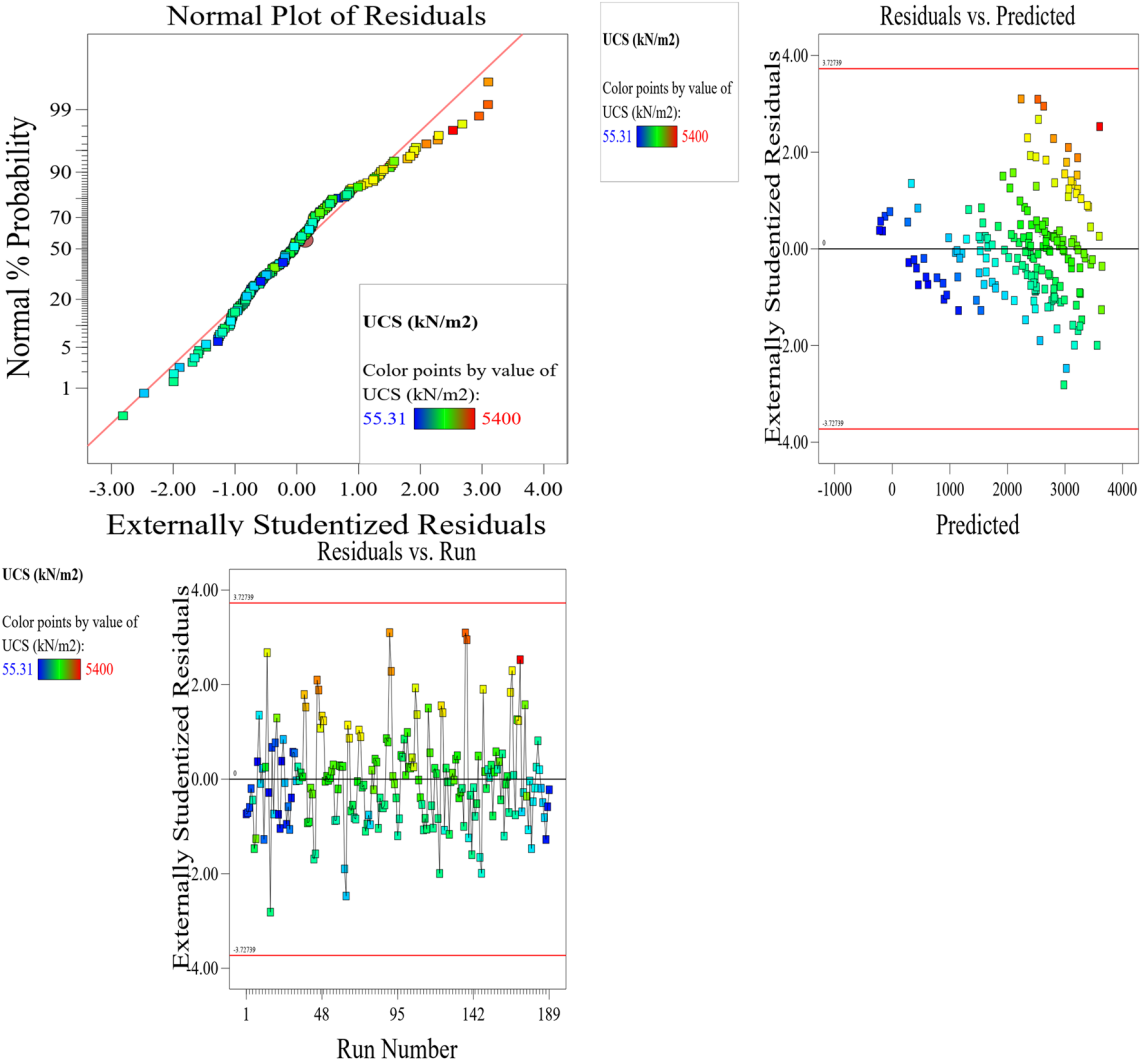
Normal plot of residuals, residuals versus predicted values and residuals versus model entry runs.

**Figure 30:**
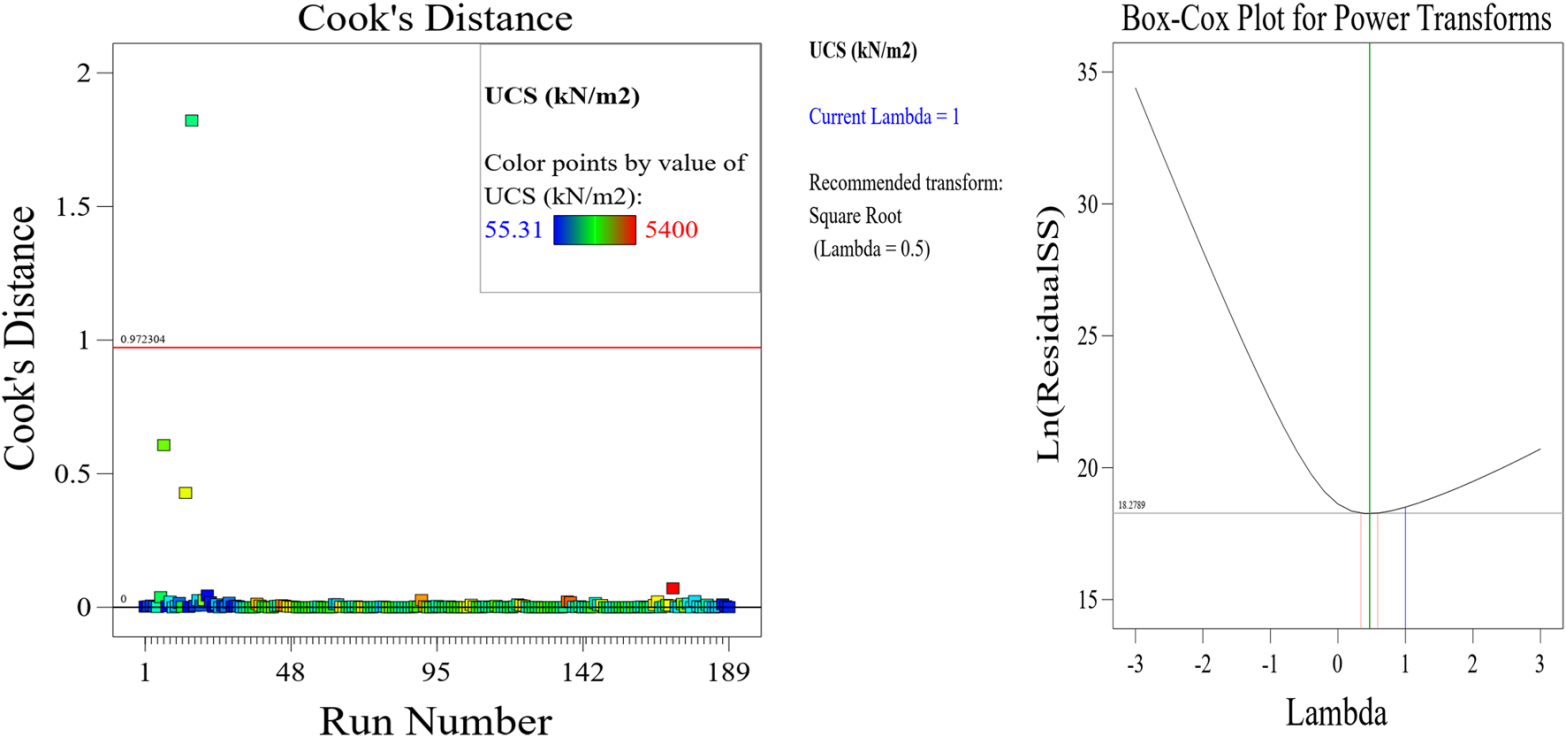
Plot of Cook’s distance and Box–Cox plot for power transforms.

**Figure 31:**
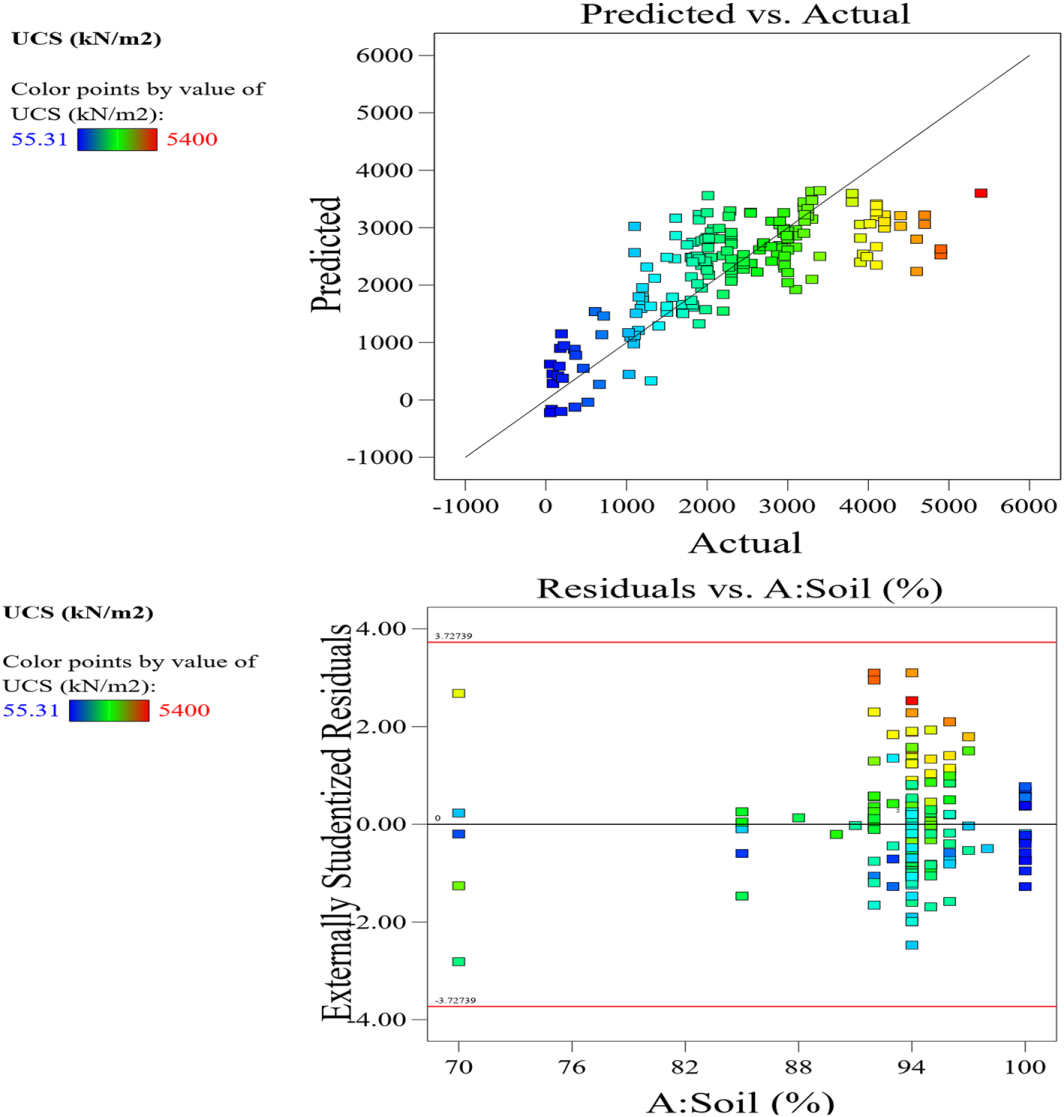
Plot of predicted versus actual UCS values and residuals versus soil proportion.

**Figure 32:**
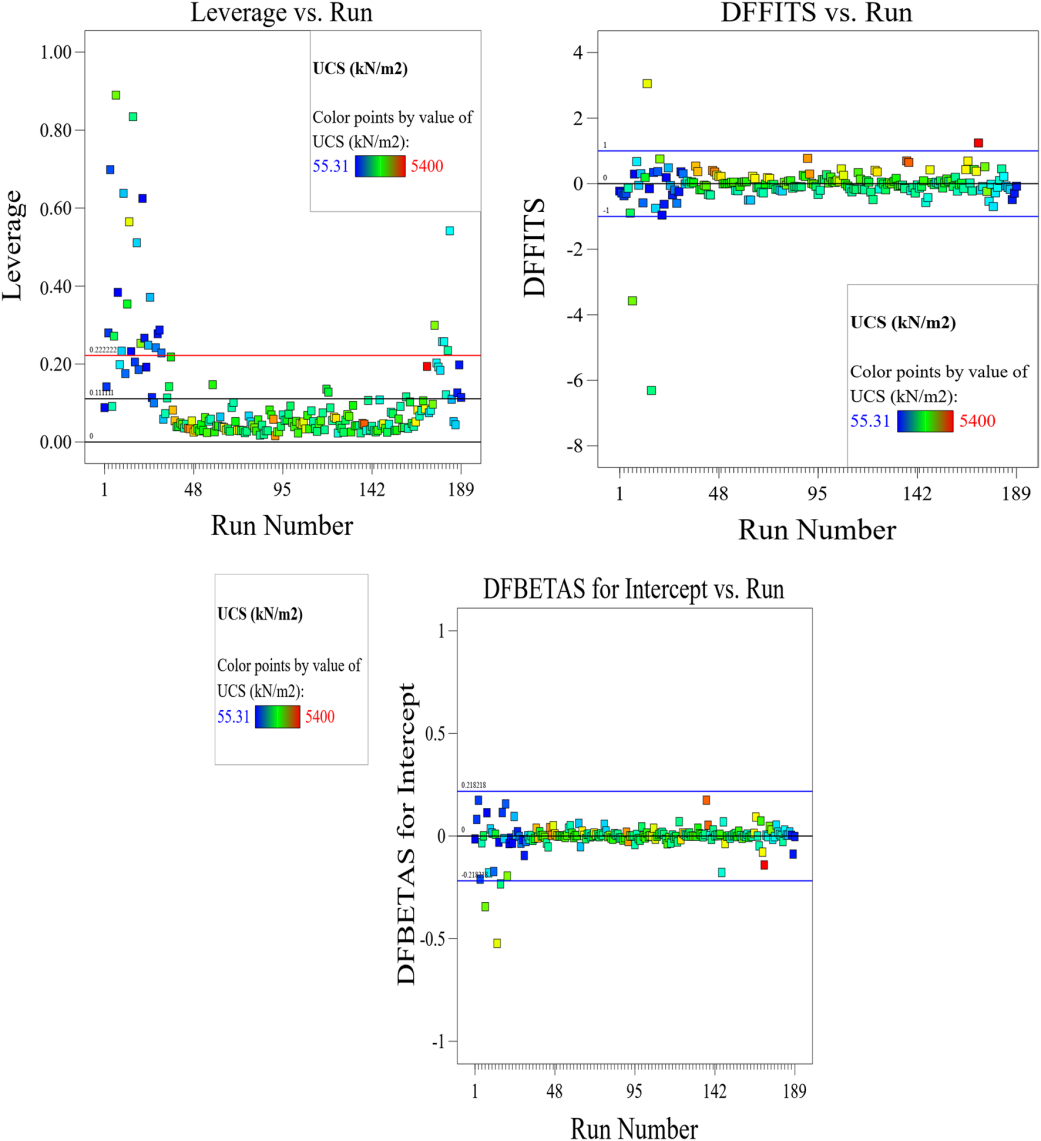
Plot of model entry runs versus leverage, DFFITS, and DFBETAS for intercept.

**Figure 33:**
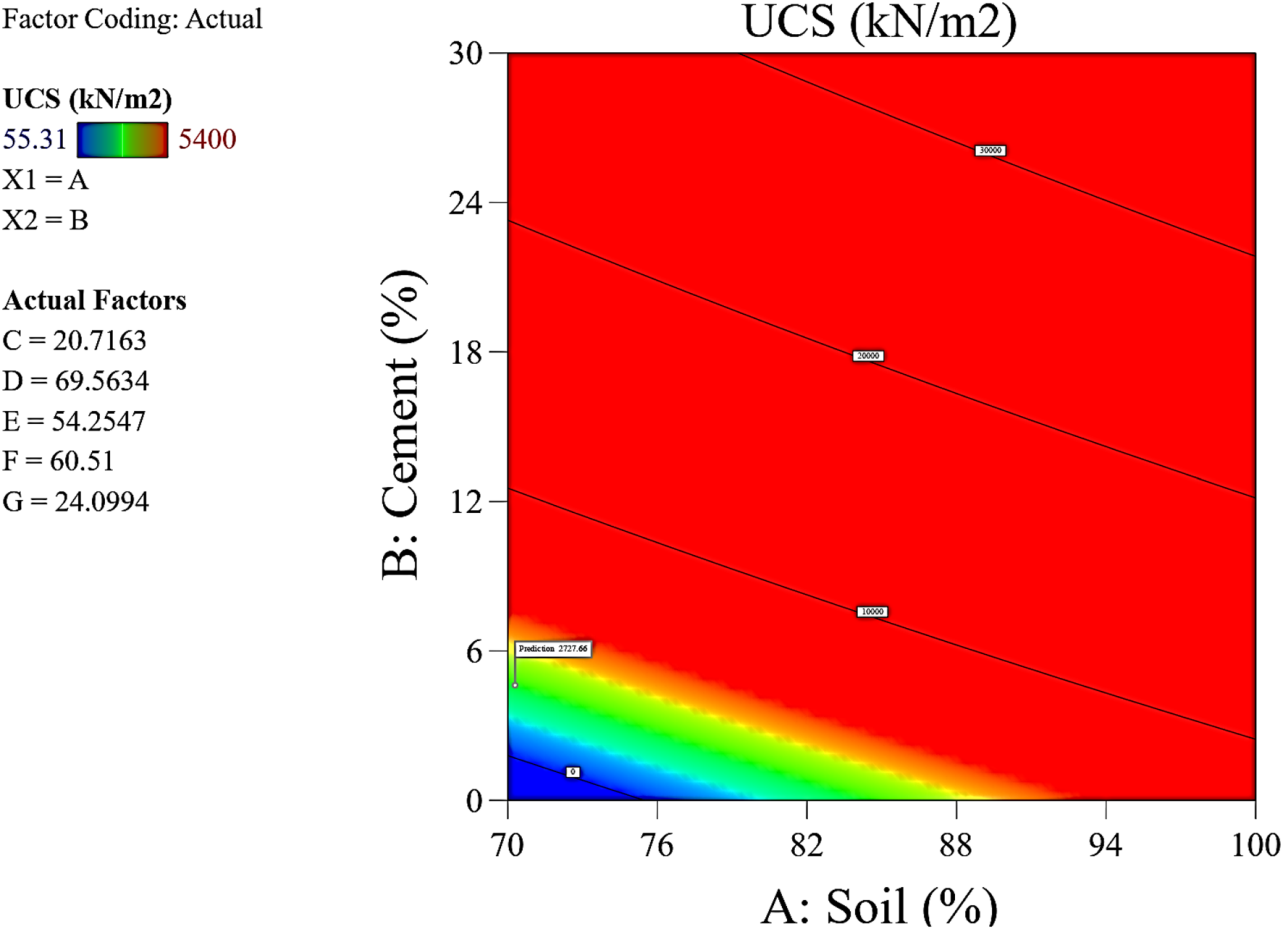
Contour plot of optimized UCS for cement and soil mix.

The Predicted R^2^ of 0.9095 is not as close to the Adjusted R^2^ of 0.9202 as one might normally expect; i.e., the difference is less than 0.2. In the case of a difference of 0.2 and more, it may indicate a large block effect or a possible problem with your model and/or data. Things to consider in such a situation are model reduction, response transformation, outliers, etc. All empirical models should be tested by doing confirmation runs. Adeq Precision measures the signal to noise ratio. A ratio greater than 4 is desirable. Your ratio of 14.398 indicates an adequate signal. This model can be used to navigate the design space. These statistical results are shown in Table 4. The graphical representation of the; normal plot of residuals, residuals versus predicted values and residuals versus model entry runs, Cook’s distance and Box-Cox plot for power transforms, predicted versus actual UCS values and residuals versus soil proportion, model entry runs versus leverage, DFFITS, and DFBETAS for intercept and the contour plot of optimized UCS for cement and soil mix are illustrated in Figs. 29, 30, 31, 32 and 33. The color points utilized in these presentations show how the UCS is optimized for the design of structures utilizing cement-lime reconstituted cohesive soils.

**Table 4:** Fit statistics of the RSM model.

Std. dev.	805.19	R^2^	0.8713
Mean	2313.99	Adjusted R^2^	0.9202
C.V. %	34.80	Predicted R^2^	0.9095
		Adeq Precision	14.3982

Overall, the present results from the multiple machine learning models compare well with the behavior of the previously reported results from the literature^2,3,13,15,16,19,21^ and this confirms the materials behavior of cement-lime reconstituted soils also reported in the literature^4,9,11^. This shows the validity of the models discussed in the paper in the sustainable design of soils treated with cement and lime based on their unconfined compressive strength. Moreso, the application of the ensemble methods in this paper has shown a more decisive outcome than reported in the previous reports as the performance shows the superiority of the incorporation of interfaces of the conventional methods and certain metaheuristic-based training algorithms.

### Sensitivity of the input variables with the output

Accordingly, the (SI) values are (0.91, 0.44, 0.73, 0.56, 0.03, 0.96) for (C), (Li), (LL), (PI), (OMC) and (MDD) respectively. A sensitivity index of 1.0 indicates complete sensitivity, a sensitivity index less than 0.01 indicates that the model is insensitive to changes in the parameter. This shows that the MDD has the highest influence on the behavior of the cement-lime reconstituted cohesive soil studied in the research project and should be considered as a decisive variable when optimal compaction of reconstituted soil is required in the construction of pavement subgrade and landfill liner facilities requiring a minimum of 200 kN/m^2^ of strength. Cement and the index properties follow in the level of importance.

## Conclusions

This research presents a comparative study between eight ensemble-based ML classification techniques namely GB, CN2, NB, SVM, SGD, K-NN, Tree and RF and the ANN and RSM to estimate the (UCS, MPa) of cohesive soil stabilized with cement and lime. The considered inputs were C, Li, LL, PI, OMC, and MDD. The outcomes of this study could be concluded as follows:

- Both (GB) and (K-NN) models showed the same excellent accuracy of 95%, while (CN2), (SVM) and (Tree) models shared the same level of accuracy of about 90%. (RF) and (SGD) models showed fair accuracy level of about 65–80% and finally (NB) presented an unacceptable low accuracy of 13%.
- The ANN and the RSM also showed closely matched accuracy to the SVM and the Tree.
- Both of correlation matrix and sensitivity analysis indicated that (UCS) is greatly affected by (MDD) then the consistency limits and cement content, and lime content comes in the third place while the impact of (OMC) is almost neglected.
- All the developed models are too complicated to be used manually, which may be considered as the main disadvantage of the ML classification techniques compared with other symbolic regression ML techniques such as (GP), (RSM), and (EPR).
- The developed models are valid within the considered range of parameter values, beyond this range; the prediction accuracy should be verified.

## Data Availability

The supporting data for this research project is available from the corresponding author on reasonable request.
